# Supplementary material for: Enzymatic synthesis of key RNA therapeutic building blocks using simple phosphate donors
Source: Nat Commun. 2025 Dec 17;17:622. doi: 10.1038/s41467-025-67366-4 (PMC12816578; doi:10.1038/s41467-025-67366-4)
Supplement: Supplementary file 1 — Supplementary Information [file 41467_2025_67366_MOESM1_ESM.pdf]

An Enzyme Cascade for Production of Key RNA Therapeutic Building Blocks Using Simple Phosphate Donors

Supplementary information

Chemical synthesis

General Information: All chemicals were purchased from commercial suppliers and were used without further purification unless otherwise stated. The use of NH_4OH refers to NH_4OH (35% w/v). Anhydrous MeCN was obtained by drying MeCN over 3 Å molecular sieves. Other anhydrous solvents were purchased from suppliers and used according to relevant guidelines. Unless otherwise stated, all reactions were conducted using anhydrous solvents, under an atmosphere of N_2 which was passed through a Drierite® drying column. Thin layer chromatography (TLC) was performed using pre-coated 0.25 mm 60 F254 silica gel plates (Merck). Flash column chromatography was performed using silica gel, high purity grade, pore size 60 Å, 230-400 mesh particle size, 40-63 µm particle size (Sigma Aldrich). NMR spectra were recorded on a Bruker Avance 400 spectrometer. The chemical shift data for ^{1}H and ^{13}C signals are given as δ in units of parts per million (ppm) relative to H_2O, where δ = 4.79 ppm or $CDCl_3$, where δ = 7.28. The number of protons (n) for a given resonance is indicated by nH. The multiplicity of each signal is indicated by: s (singlet), br s (broad singlet), d (doublet), t (triplet), q (quartet), m (multiplet) or variations thereof. Coupling constants (*J*) are quoted in Hz and calculated to the nearest 0.1 Hz.

General procedure for the purification of nucleotide phosphates on silica gel. In the purification of **1b** and **1d** on silica gel, a column without a frit was pAcKed first with cotton wool followed by a thin layer of sand, a filter paper, and finally with silica gel in ^{i}PrOH. The crude material was dissolved in a minimum amount of H_2O or ^{i}PrOH and loaded onto the column. The column was eluted first with ^{i}PrOH, then 95:5 ^{i}PrOH/NH_4OH, gradually increasing the amount NH_4OH up to 1:1 ^{i}PrOH/NH_4OH. Fractions containing the product (confirmed *via* TLC analysis, 3:2 ^{i}PrOH/NH_4OH) were pooled and concentrated *in vacuo*. The purified material was then subject to Na^+ ion exchange (using resin; DOWEX 50W X8, Na^+ form) to afford the sodium salt of the product.

Preparation of 1b (2'-MOE-AMP). To an oven-dried two-necked round bottom flask equipped with a magnetic stirrer bar, **1a** (200 mg, 0.61 mmol, 1.0 equiv.) was added, and the reaction flask was evacuated and refilled with N_2 three times. **1a** was suspended in $PO(OMe)_3$ (2.4 mL), and the mixture was cooled to −20 °C (ice/NaCl bath). $POCl_3$ (68 µL, 0.73 mmol, 1.2 equiv.) was added dropwise, and the reaction mixture was allowed to warm to −10 °C and maintained between −10 and 0 °C for 4 h, at which point TLC analysis (3:2 ^{i}PrOH/NH_4OH) indicated complete consumption of the starting material to a lower R_f spot. The reaction was quenched with H_2O (2.0 mL), and the mixture stirred for a further 30 min at 0 °C before being washed with DCM (3 x 15 mL), and the remaining phase concentrated *in vacuo* at ≤30 °C to a crude residue. The crude residue was purified *via* flash column chromatography on silica gel (0–30% NH_4OH/^{i}PrOH), and the appropriate fractions pooled and concentrated *in vacuo* at ≤30 °C. The residue was converted to the corresponding sodium salt and the sample freeze dried to give the title compound (261 mg, 0.58 mmol, 95%) as a white solid. R_f = 0.32 (3:2, ^{i}PrOH/NH_4OH); ^{1}H NMR (400 MHz, D_2O): δ 8.42 (1H, s), 8.09 (1H, s), 6.10 (d, *J* = 5.8 Hz), 4.60 (1H, dd, *J* = 5.0, 3.5 Hz), 4.50 (1H, app. t, *J*, = 5.4 Hz), 4.41–4.36 (1H, m), 4.20–4.10 (2H, m, H), 3.83–3.77 (1H, m), 3.73–3.66 (1H, m), 3.55–3.43 (2H, m), 3.16 (3H, s); ^{13}C NMR (101 MHz, D_2O) δ 154.6, 151.9, 148.6, 140.0, 118.2, 85.7, 84.4 (d, *J* = 8.6 Hz), 82.4, 71.1, 69.6, 69.4, 64.5 (d, *J* = 5.0 Hz), 58.0; ^{31}P{^{1}H} NMR (162 MHz, D_2O): δ 0.45 (s); HRMS (NSI): calculated for $C_{13}H_{17}N_5O_8PNa_2$ $[M-H]^-$ 448.0616, found 448.0615.

Preparation of Bis(tributylammonium)pyrophosphate. $Na_4P_2O_7 \cdot 10H_2O$ (685 mg, 1.54 mmol, 1.0 equiv.) was dissolved in a minimal amount of H_2O and loaded onto a H^+ resin column (DOWEX

50W 8X, H^+ form). The column was eluted with H_2O directly into a round-bottom flask containing Bu_3N (731 µL, 3.08 mmol, 2.0 equiv.) in EtOH (3.1 mL) at 0 °C, until the eluent was pH ~7. The mixture was concentrated *in vacuo*, and the residue was transferred to a Falcon™ tube and freeze dried to give the title compound (844 mg, 1.54 mmol, quant.) as an off-white gum.

Preparation of 1d (2'-MOE-ATP). To an oven-dried two-necked round bottom flask equipped with a magnetic stirrer bar, **1a** (250 mg, 0.77 mmol, 1.0 equiv.) was added, and the reaction flask was evacuated and refilled with N_2 three times. **1a** was suspended in $PO(OMe)_3$ (1.0 mL), and the mixture was cooled to −20 °C (ice/NaCl bath). $POCl_3$ (86 µL, 0.73 mmol, 1.2 equiv.) was added dropwise, and the reaction mixture was allowed to warm to −10 °C and maintained between −10 and 0 °C for 2 h, at which point TLC analysis (3:2 ^{i}PrOH/NH_4OH) indicated complete consumption of the starting material to a lower R_f spot. The reaction was cooled to −10 °C and a precooled (−20 °C) solution of bis(tributylammonium)pyrophosphate (844 mg, 1.54 mmol, 2.0 equiv.) in MeCN (3.9 mL) and Bu_3N (1.1 mL, 4.62 mmol, 6.0 equiv.) was added in one portion. The reaction mixture was then allowed to slowly warm to 0 °C over 1 h, at which point TLC analysis (2:3 ^{i}PrOH/NH_4OH) indicated complete consumption of the monophosphate intermediate to a lower R_f spot. The reaction was quenched with H_2O (2 mL) and the mixture stirred for a further 1.5 h at 0 °C before being washed with DCM (3 x 15 mL), and the remaining phase concentrated *in vacuo* at ≤30 °C to give a crude residue. The crude residue was purified *via* flash column chromatography on silica gel (0–50% NH_4OH/^{i}PrOH), and the appropriate fractions were pooled and concentrated *in vacuo* at ≤30 °C. The residue was converted to the corresponding sodium salt, and the sample freeze dried to give the title compound as a white solid. R_f = 0.14 (2:3, ^{i}PrOH/NH_4OH); ^{1}H NMR (400 MHz, D_2O): δ 8.52 (1H, s), 8.24 (1H, s), 6.20 (1H, d, *J* = 5.9 Hz), 4.71 (1H, dd, *J* = 5.2, 3.4 Hz), 4.60 (1H, app. t, *J* = 5.6 Hz), 4.47–4.39 (1H, m), 4.35–4.17 (2H, m), 3.86 (1H, ddd, *J* = 11.8, 5.7, 3.1 Hz), 3.74 (1H, ddd, *J* = 11.8, 5.5, 3.3 Hz), 3.59–3.45 (2H, m), 3.17 (3H, s); ^{13}C NMR (101 MHz, D_2O): δ 155.6, 152.8, 149.0, 140.0, 118.5, 85.5, 84.4 (d, *J* = 9.2 Hz), 82.2, 71.0, 69.6, 69.0, 65.1 (d, *J* = 5.9 Hz), 57.9; ^{31}P NMR (162 MHz, D_2O): δ −7.03 (br. s), −10.9 (d, *J* = 18.9 Hz), −21.6 (br. S); HRMS (NSI) *m/z* calculated for $C_{13}H_{18}N_5Na_3O_{14}P_3$ $[M{-}Na]^-$ 629.9762, found 629.9764.

Preparation of 27a. A solution of 1-((2R,4S,5R)-5-((bis(4-methoxyphenyl)(phenyl)methoxy)methyl)-4-hydroxytetrahydrofuran-2-yl)-5-methylpyrimidine-2,4(1H,3H)-dione (272 mg, 0.5 mmol, 1.0 eq.), allyl bromide (90 µL, 1.0 mmol, 2.0 eq.) and NaOH (270 mg, 6.75 mmol, 13.0 eq.) in toluene (3 mL) was heated to 80 °C for 5 h. The reaction was cooled to room temperature and the solids were removed by filtration. The filtrate was concentrated to yield the crude product, which was used in the following step without further purification. To a solution of 1-((2R,4S,5R)-4-(allyloxy)-5-((bis(4-methoxyphenyl)(phenyl)methoxy)methyl)tetrahydrofuran-2-yl)-5-methylpyrimidine-2,4(1H,3H)-dione in CH_2Cl_2 (40 mL) was added TFA (1 mL) at 0 °C. The reaction was stirred for 1 h and then sat. aq. $NaHCO_3$ (20 mL) was added. The layers were separated and the aqueous layer was washed with CH_2Cl_2 (3 x 15 mL), the combined organics were dried ($MgSO_4$) and concentrated. The crude product was purified by silica column (CH_2Cl_2/MeOH 97:3 to 9:1) to yield the title product as a white solid (42 mg, 0.15 mmol, 30%). ^{1}H NMR (400 MHz, $CDCl_3$) δ 8.62 (1H, s), 7.37 (1H, d, *J* = 1.5 Hz), 6.12 (1H, t, *J* = 7.0 Hz), 6.01 – 5.83 (1H, m), 5.32 (1H, dd, *J* = 17.2, 1.5 Hz), 5.24 (1H, dd, *J* = 10.4, 1.5 Hz), 4.26 (1H, dt, *J* = 6.5, 3.5 Hz), 4.13 (1H, q, *J* = 3.0 Hz), 4.11 – 3.92 (3H, m), 3.85 – 3.76 (1H, m), 2.63 (1H, s), 2.43 – 2.34 (2H, m), 1.93 (3H, d, *J* = 1.2 Hz); ^{13}C NMR (101 MHz, $CDCl_3$) δ 163.6, 150.3, 137.1, 134.1, 117.6, 111.1, 87.5, 85.2, 78.5, 70.5, 62.8, 37.1, 12.5 ppm.

Preparation of 28a. To a solution of 1-((2*R*,4*S*,5*R*)-5-((bis(4-methoxyphenyl)(phenyl)methoxy)methyl)-4-hydroxytetrahydrofuran-2-yl)-5-methylpyrimidine-2,4(1*H*,3*H*)-dione (272 mg, 0.5 mmol, 1.0 eq.), 4-dimethylaminopyridine (31 mg, 0.125 mmol, 0.25 eq.) and triethylamine (0.17 mL, 1.25 mmol, 2.5 eq.) in pyridine (6 mL) was added acetic

anhydride (85 μL, 0.75 mmol, 1.5 eq.) dropwise at 0 °C. The reaction was stirred at room temperature for 1 h and then quenched by addition of sat. aq. $NaHCO_3$ (20 mL). The mixture was extracted with EtOAc (3 x 15 mL), the organics were dried ($MgSO_4$) and concentrated. The crude product was used in the following step without further purification. To a solution of (2*R*,3*S*,5*R*)-2-((bis(4-methoxyphenyl)(phenyl)methoxy)methyl)-5-(5-methyl-2,4-dioxo-3,4-dihydropyrimidin-1(2*H*)-yl)tetrahydrofuran-3-yl acetate (0.5 mmol, 1.0 eq.) in CH_2Cl_2 (40 mL) was added TFA (1 mL) at 0 °C. The reaction was stirred for 1 h and then sat. aq. $NaHCO_3$ (20 mL) was added. The layers were separated and the aqueous layer was washed with CH_2Cl_2 (3 x 15 mL), the combined organics were dried ($MgSO_4$) and concentrated. The crude product was purified by silica column (CH_2Cl_2/MeOH 98:2 to 9:1) to yield the title product as a white solid (68 mg, 0.24 mmol, 48%). ^{1}H NMR (400 MHz, $CDCl_3$) δ 8.22 (1H, br s), 7.51 (1H, d, *J* = 1.2 Hz), 6.27 (1H, dd, *J* = 8.2, 6.1 Hz), 5.37 (1H, dt, *J* = 5.8, 2.6 Hz), 4.12 (1H, q, *J* = 2.6 Hz), 4.01 – 3.88 (2H, m), 2.50 – 2.36 (2H, m), 2.32 (1H, br s), 2.13 (3H, s), 1.96 (3H, d, *J* = 1.2 Hz); ^{13}C NMR (101 MHz, $CDCl_3$) δ 170.7, 163.2, 150.2, 136.2, 111.4, 86.0, 85.0, 74.7, 62.7, 37.2, 21.0, 12.6 ppm.

Supplementary Figures and Tables

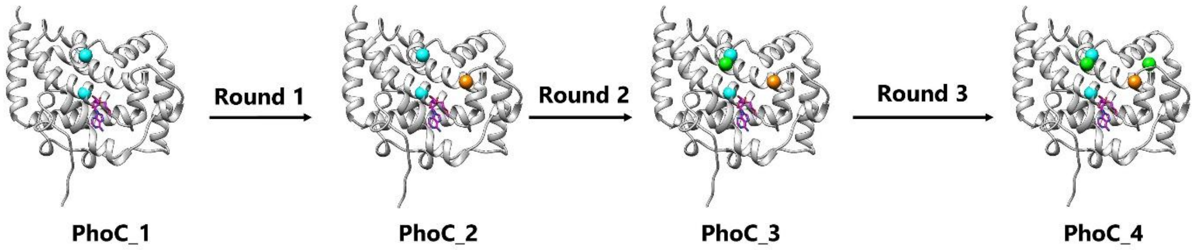

Round	Description	Theoretical library size	Clones screened	Beneficial mutations	Best variant
1	Saturation mutagenesis of active site residues	640	1800	K31C, L34E, P51Q, R71S, R74S, A82S, A86E, E122C, D154L, G167W	PhoC_2 = PhoC_1[1] + D154L
2	Saturation mutagenesis of active site, second coordination sphere and flexible loop residues	640	1800	A90E, A90V, N151A	PhoC_3 = PhoC_2 + A90E
3	Combination of Round 2 beneficial mutations	2	2		PhoC_4 = PhoC_3 + N151A

[1] PhoC 1 = Non-specific acid phosphatase from *Morganella morganii* + G92D, I171T mutations

Supplementary Fig. 1. Directed evolution of PhoC_1. Structures showing the amino acid positions mutated throughout evolution (represented as spheres). PhoC_1 contains G92D and I171T mutations (shown in cyan). Three mutations were installed during the course of evolution, D154L (orange), A90E and N151A (green). The table describes the method of library generation used, the number of clones evaluated and the sequence of the most improved variant for each round.

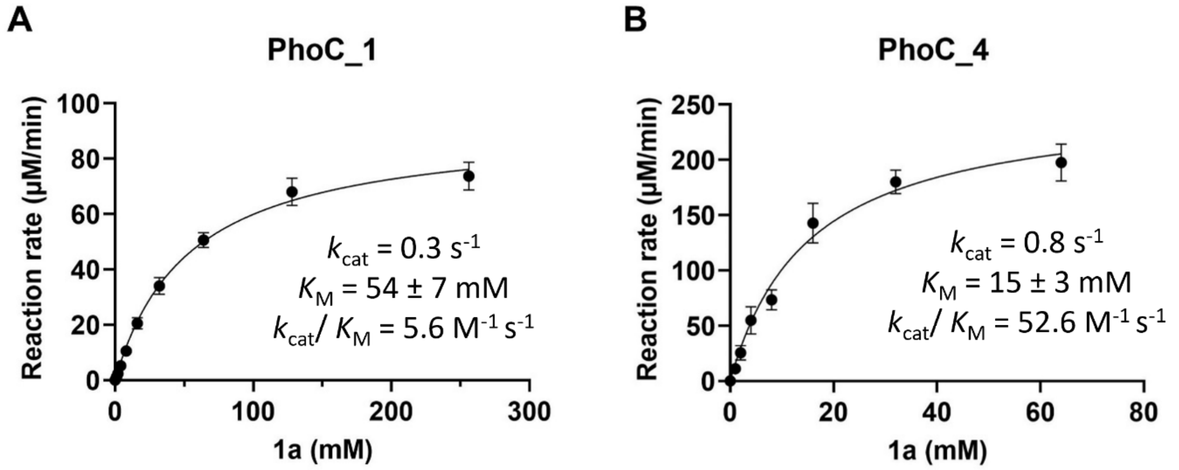

Supplementary Fig. 2. Kinetic characterization of PhoC_1 and PhoC_4. Michaelis–Menten plots of the phosphorylation of 2'-MOE-A (**1a**) by **A)** PhoC_1 and **B)** PhoC_4. Kinetic assays were performed at a fixed concentration of pyrophosphate (200 mM). The plots show averaged initial rates which were fitted to the Michalis-Menten equation. Data are mean ± s.d. of measurements made in triplicate. Source data is provided in the source data file.

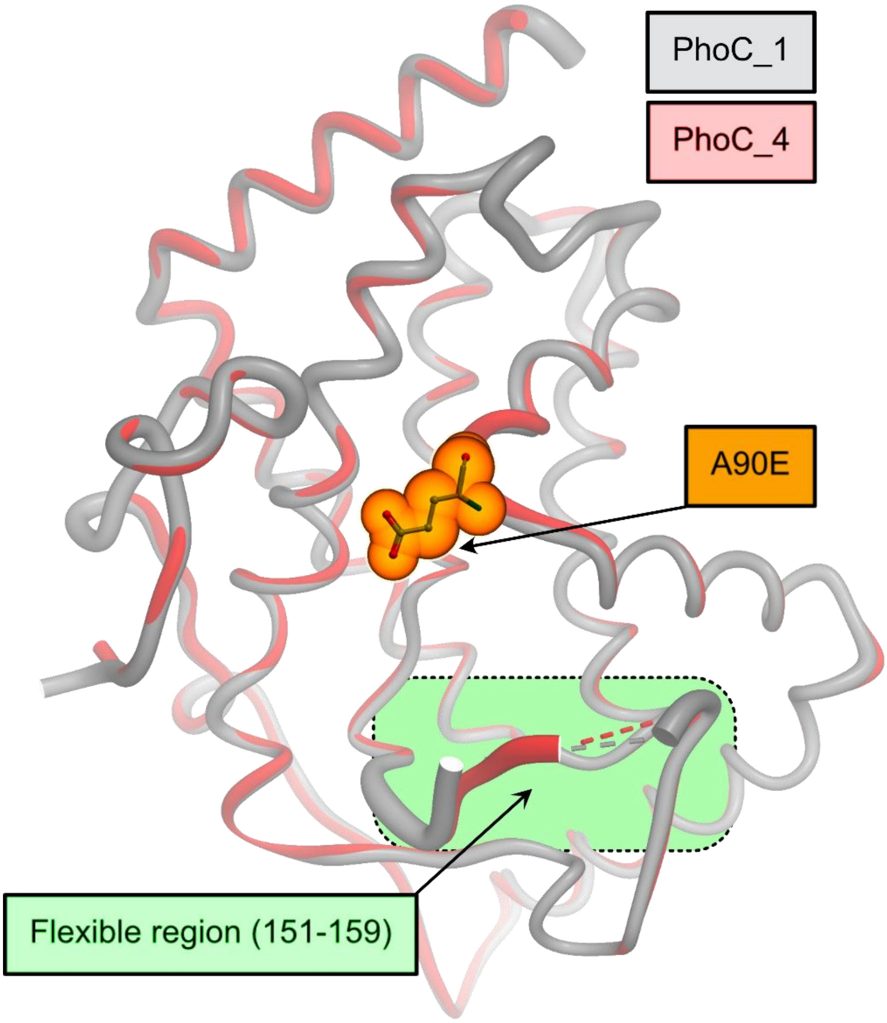

Supplementary Fig. 3. Structural Characterization of PhoC_1 and PhoC_4. A superposition of PhoC_1 (grey) and PhoC_4 (red) is shown in protein worm representation scaled by B-factor. The location of A90E is highlighted in ball and stick representation and associated CPK spheres (orange). The flexible loop region, residues 151-159, is highlighted in green.

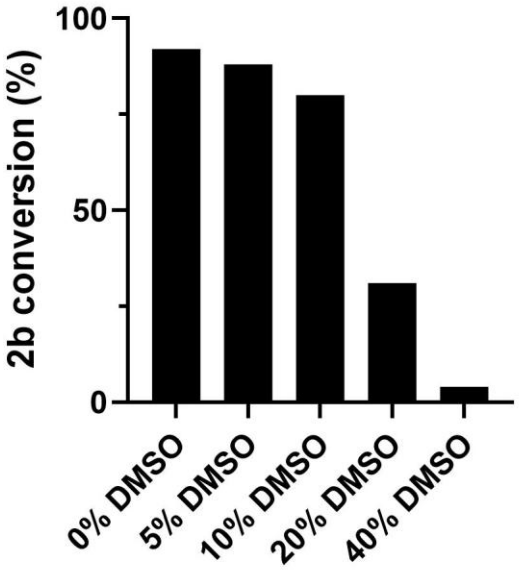

Supplementary Fig. 4. Activity of PhoC_4 in the presence of DMSO. Percentage conversion of adenosine (**2a**) to AMP (**2b**) product. Biotransformations were performed using **2a** (1 mM), pyrophosphate (200 mM), PhoC_4 (20 μM), and 0-40% DMSO in 20 mM sodium acetate buffer pH 4 at 30 °C. Source data is provided in the source data file.

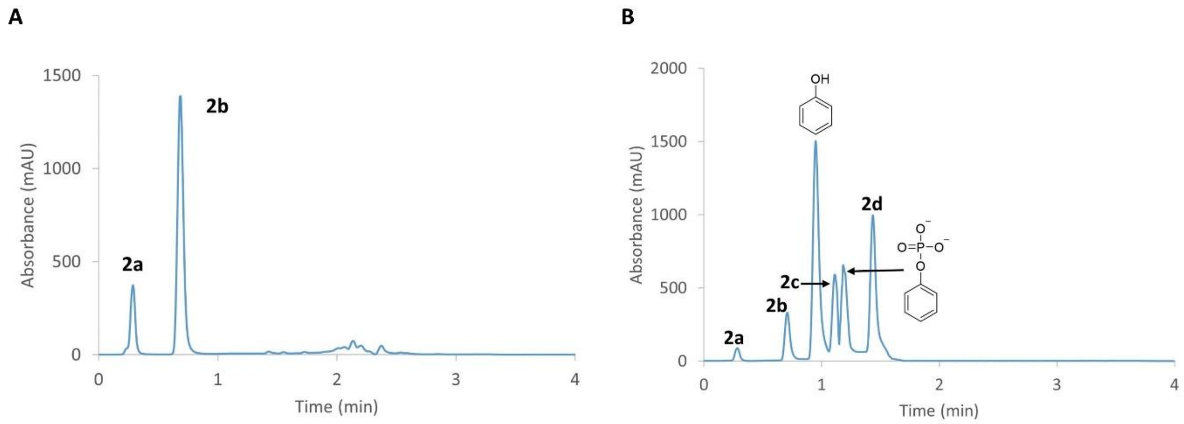

Supplementary Fig. 5. **One-pot PhoC_4 and PPK5 cascade reactions.** HPLC traces showing the activity of PhoC_4 (20 μM) and PPK5 (30 μM) towards adenosine (**2a**,10 mM) in the presence of 20 mg/mL hexametaphosphate, 50 mM $MgCl_2$, 20 mM sodium acetate, 50 mM Tris-HCl, pH 5 and **A)** pyrophosphate (200 mM) or **B)** phenylphosphate (200 mM). Pyrophosphate inhibits PPK activity and only the AMP (**2b**) intermediate is detected.

^{1}H NMR (400 MHz, D_2O)

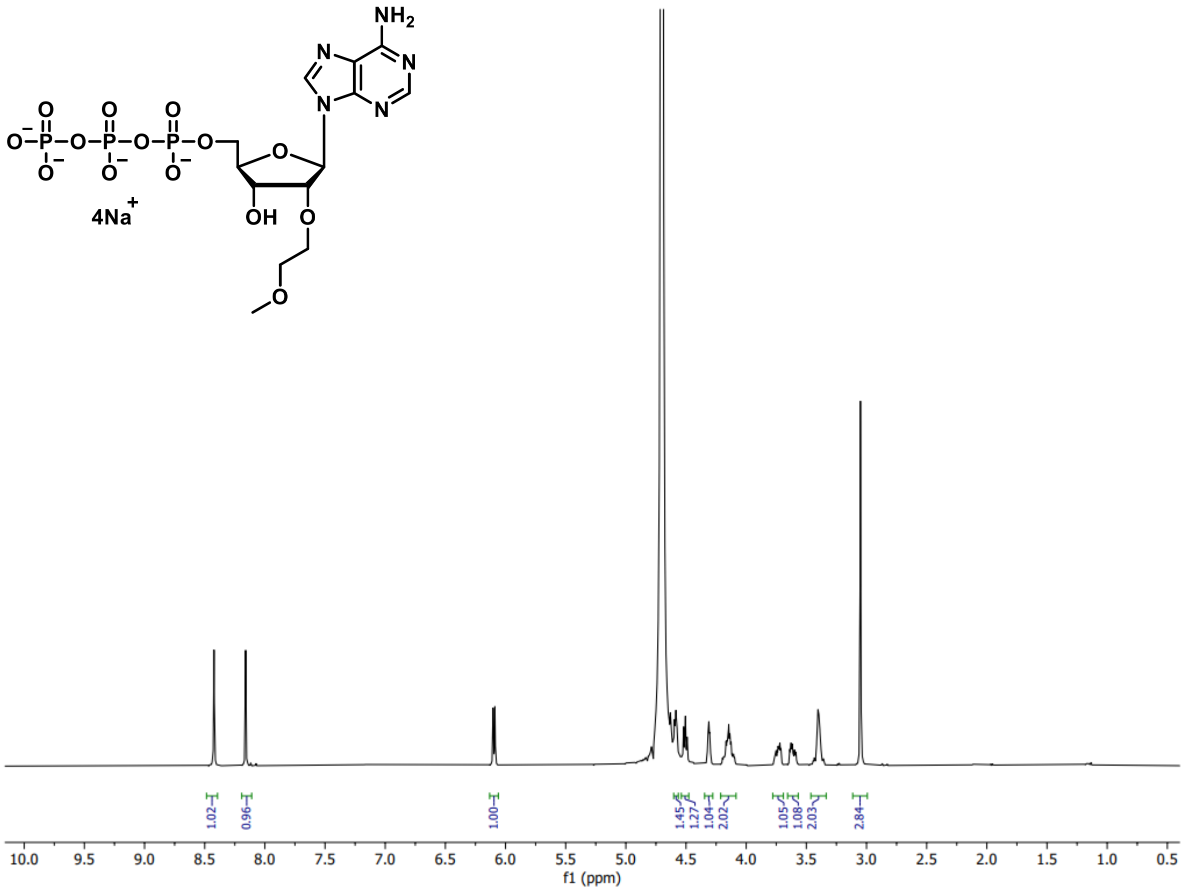

^{31}P NMR (162 MHz, D_2O)

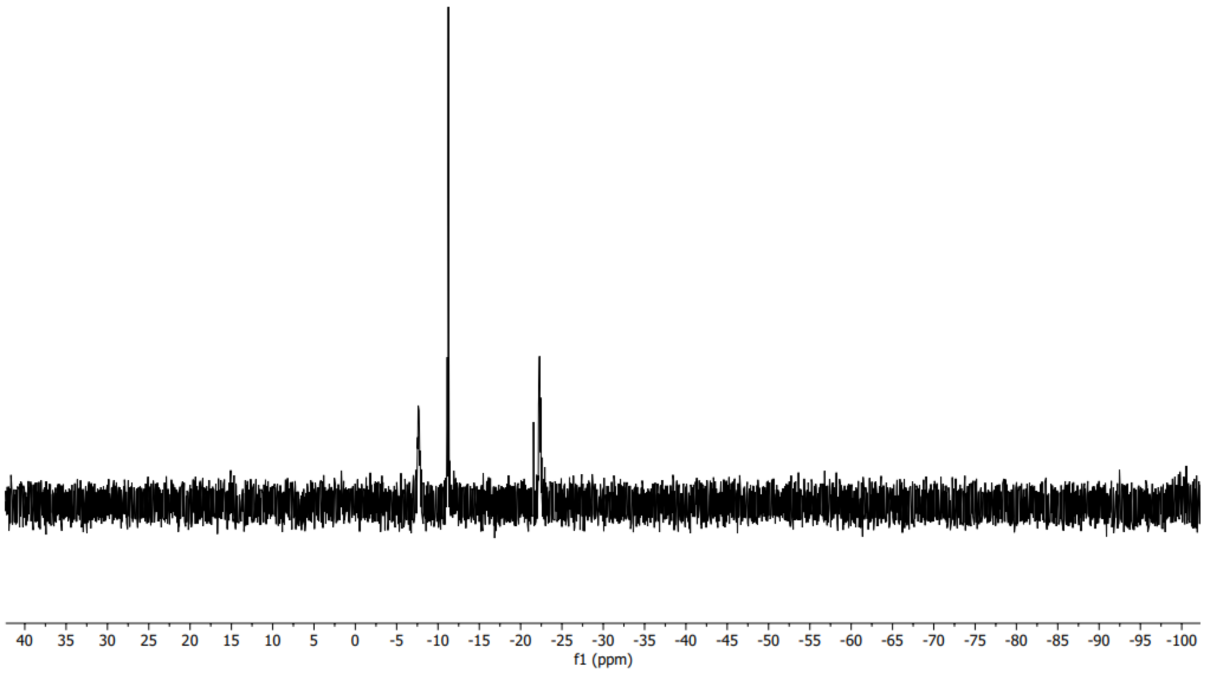

Supplementary Fig. 6. NMR spectra of enzymatically synthesized 2’-MOE-ATP (1d)

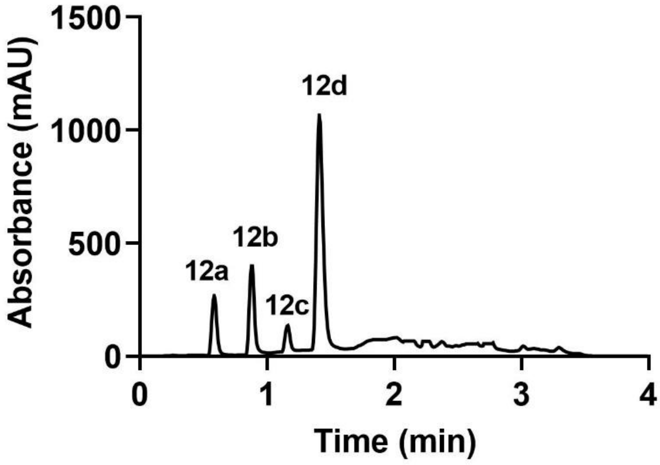

Supplementary Fig. 7: Enzymatic synthesis of 2'F-ATP (12d). HPLC trace showing enzymatic 2'F-ATP synthesis on a 500 mg scale. Impurities include 2'F-A (**12a**), 2'F-AMP (**12b**) and 2'F-ADP (**12c**). The trace shows the crude reaction mixture that was used in enzymatic oligonucleotide synthesis without further purification.

	Exp. mass (M-H⁺)	Obs. mass	Sequence
P	2666.6	2666.5	$(2'F\text{-}A)_8$
a	2997.8	2997.4	$(2'F\text{-}A)_9$
b	2335.4	2336.1	$(2'F\text{-}A)_7$
c	2004.2	2004.9	$(2'F\text{-}A)_6$
d	2664.6	2664.2	$(2'F\text{-}A)_7 + A_{OH}$
e	2995.8	2995.9	$(2'F\text{-}A)_8 + A_{OH}$
f	2333.4	2334.0	$(2'F\text{-}A)_6 + A_{OH}$
g	2002.2	2002.8	$(2'F\text{-}A)_5 + A_{OH}$

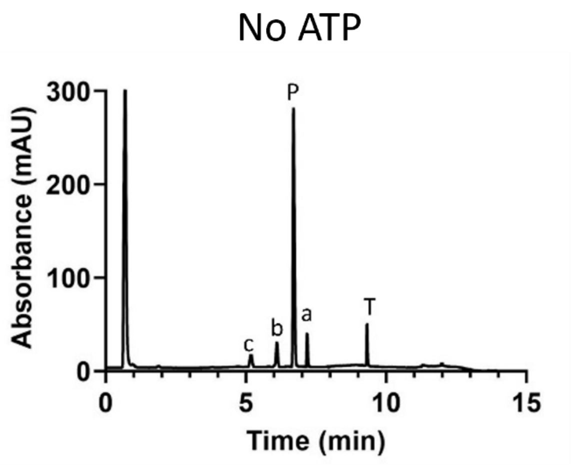

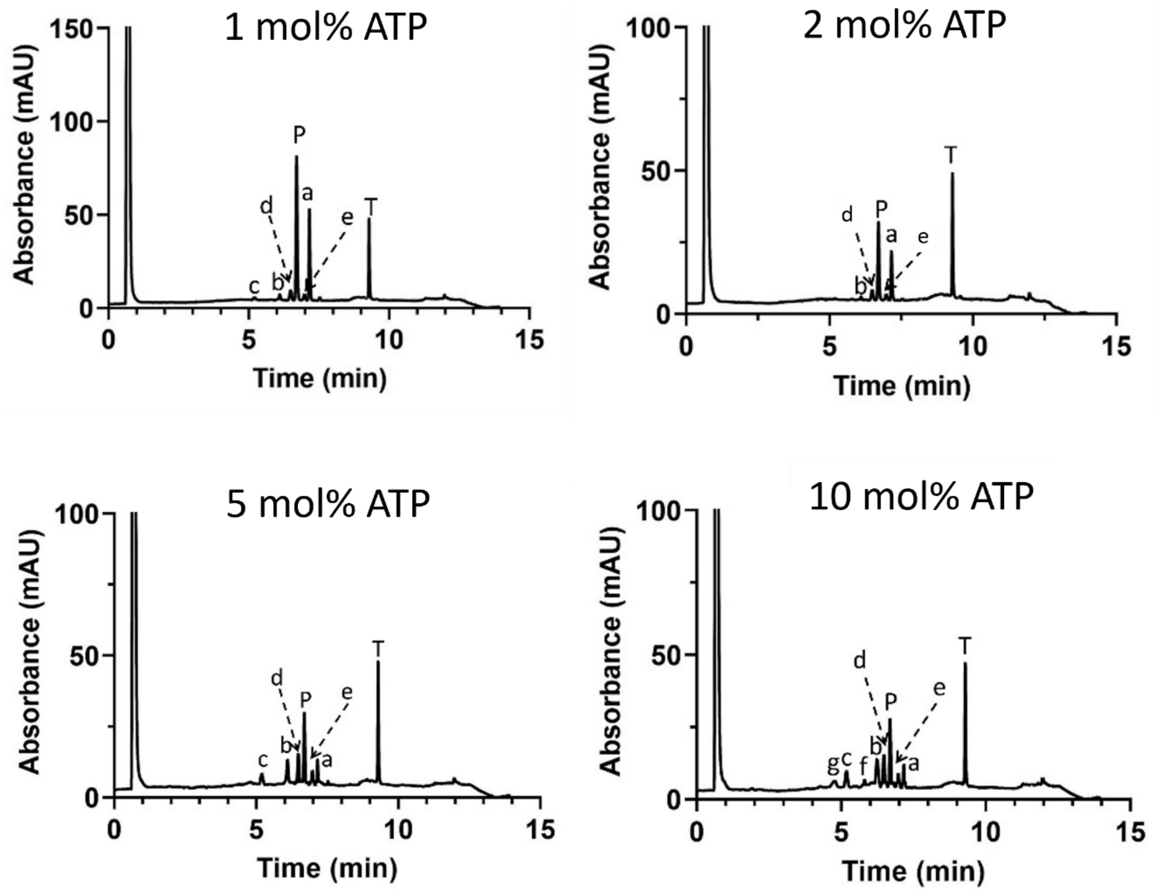

Supplementary Fig. 8: Enzymatic synthesis of a poly-2'-F-A 8mer in the presence of ATP impurities. 2'-F-ATP produced using our ATP-free enzyme cascade was used in crude form directly in biocatalytic oligonucleotide synthesis (No ATP). Spiking the 2'-F-ATP substrates with 1-10 mol% ATP impurities results in complex product distributions arising from competing ATP and 2'-F-ATP incorporation.

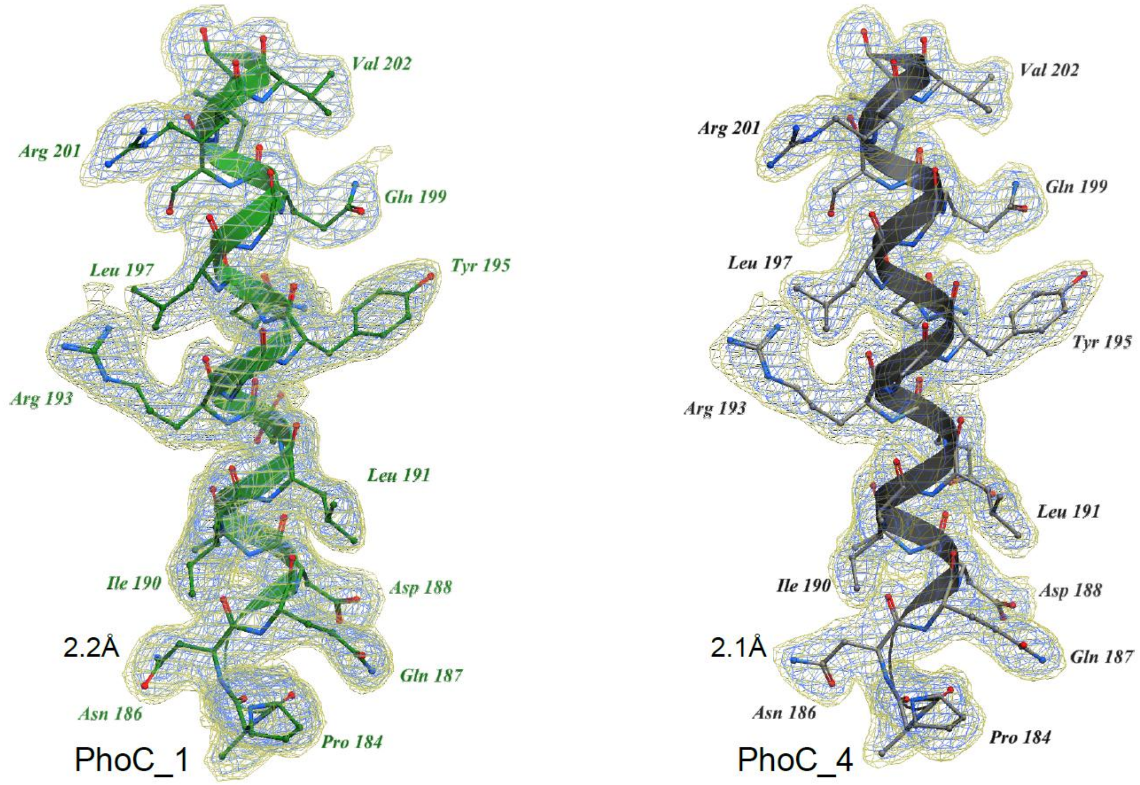

Supplementary Fig. 9. Representative electron density map for PhoC_1 and PhoC_4. Representative 2Fo-Fc electron density contoured at 1.0σ (yellow) and 2.5σ (blue) for the crystal structures of PhoC_1 (2.2Å) and PhoC_4 (2.1Å). Residues 184-203 are shown for both structures (PhoC_1 in green & PhoC_4 in grey), ribbon and all atom-coloured ball and stick representations are displayed.

Supplementary Table 1. PhoC_4 biotransformations performed at high substrate loadings. Reaction conditions: nucleoside (25-100 mM), pyrophosphate (200 mM) and enzyme (20 μM) in 20 mM sodium acetate buffer pH 4 at 30 °C. [a] Due to low solubility of nucleoside **29a** the reaction was performed as a slurry.

**Substrate**	**[substrate] (mM)**	**DMSO (%)**	**Conversion to NMP (%)**	**Reaction Time (h)**
A, **2a**	50	10	83	4
C, **4a**	50	0	97	4
dA**, 6a**	50	5	74	16
2'-F-A, **12a**	50	10	82	4
2'-F-C, **14a**	100	10	91	4
2'-MeO-A, **16a**	50	10	82	16
2'-MOE-A, **1a**	100	0	87	16
2'-MOE-G, **20a**	25	10	86	16
2'-MOE-T, **22a**	100	0	83	16
LNA-T, **26a**	100	0	99	4
3'-ONH_2-A **29a**	25[a]	10	61	16

Supplementary Table 2. Substrate scope of a PPK panel. Biotransformations containing NMPs (obtained from PhoC_4 mediated reactions), hexametaphosphate (20 mg/mL), $MgCl_2$ (50 mM) and PPK as lyophilized cell-free extracts (1 mg/mL) were incubated for 18 h at 30 °C. The most suitable PPK for each substrate is shown highlighted in blue.

PPK	AMP **2b**		CMP **4b**		dAMP **6b**		2'-F-AMP **12b**		2'-F-CMP **14b**		2'-MeO-AMP **16b**	
	NDP	NTP	NDP	NTP	NDP	NTP	NDP	NTP	NTP	NTP	NDP	NTP
PPK1	30	31	35	43	25	26	27	33	31	29	28	35
PPK2	35	35	16	17	33	31	26	35	7	4	33	47
PPK3	33	37	25	20	31	32	27	49	18	8	32	40
PPK4	39	42	9	5	36	42	28	46	19	10	40	40
PPK5	38	43	7	4	35	43	35	39	14	8	47	30
PPK6	39	42	27	14	38	38	32	42	46	32	48	33
PPK7	45	33	7	1	50	26	43	19	18	6	63	18
PPK8	29	7	3	0	22	5	36	16	8	1	64	6
PPK9	46	27	6	1	51	17	46	15	11	1	65	7

PPK	2'-MOE-AMP **1b**		2'-MOE-GMP **20b**		2'-MOE-TMP **22b**		LNA-TMP **26b**		3'-NH_2-AMP **29b**	
	NDP	NTP	NDP	NTP	NDP	NTP	NDP	NTP	NDP	NTP
PPK1	32	43	23	14	16	12	14	9	20	0
PPK2	35	48	35	48	19	22	8	1	27	32
PPK3	36	47	34	47	18	23	13	1	23	33
PPK4	11	7	11	5	6	3	12	5	31	30
PPK5	9	5	5	4	2	3	9	3	29	30
PPK6	36	43	25	17	40	36	32	16	26	36
PPK7	62	13	6	1	6	1	5	1	48	0
PPK8	20	3	7	1	4	1	5	1	49	0
PPK9	40	2	7	0	4	1	8	0	59	0

Supplementary Table 3. Oligonucleotide sequences used for PhoC engineering

Name	Sequence (5‘-3’)
N26NNK-F	GCCATTCCTGCGGGANNKGACGCTACGACTAAG
N26NNK-R	TCCCGCAGGAATGGCAGC
K31NNK-F	AACGACGCTACGACTNNKCCCGATCTTTATTAT
K31NNK-R	AGTCGTAGCGTCGTTTCC
L34NNK-F	ACGACTAAGCCCGATNNKTATTATCTGAAGAAC
L34NNK-R	ATCGGGCTTAGTCGTAGC
Y35NNK-F	ACTAAGCCCGATCTTNNKTATCTGAAGAACGAA
Y35NNK-R	AAGATCGGGCTTAGTCGT
A86NNK-F	CAGGCCCAGGCTGATNNKGATTTGGCTGCCGGT
A86NNK-R	ATCAGCCTGGGCCTGTTT
A89NNK-F	GCTGATGCCGATTTGNNKGCCGGTGATGTCGCC
A89NNK-R	CAAATCGGCATCAGCCTG
I121NNK-F	CTGTTGACAAACATGNNKGAAGACGCCGGGGAC
I121NNK-R	CATGTTTGTCAACAGCTT
E122NNK-F	TTGACAAACATGATCNNKGACGCCGGGGACCTG
E122NNK-R	GATCATGTTTGTCAACAG
G125NNK-F	ATGATCGAAGACGCCNNKGACCTGGCAACTCGC
G125NNK-R	AGTTGCCAGGTCCCCGGC
D126NNK-F	ATCGAAGACGCCGGGNNKCTGGCAACTCGCTCA
D126NNK-R	CCCGGCGTCTTCGATCAT
R130NNK-F	GGGGACCTGGCAACTNNKTCAGCAAAGGAACAC
R130NNK-R	AGTTGCCAGGTCCCCGGC
K133NNK-F	GCAACTCGCTCAGCANNKGAACACTATATGCGC
K133NNK-R	TGCTGAGCGAGTTGCCAG
E134NNK-F	ACTCGCTCAGCAAAGNNKCACTATATGCGCATT
E134NNK-R	CTTTGCTGAGCGAGTTGC
R140NNK-F	CACTATATGCGCATTNNKCCGTTTGCATTCTAT
R140NNK-R	AATGCGCATATAGTGTTC
D154NNK-F	ACTTGTAATACAAAANNKCAGAAGAAGCTGTCA
D154NNK-R	TTTTGTATTACAAGTCTC
K157NNK-F	ACAAAAGACCAGAAGNNKCTGTCAACCAACGGA
K157NNK-R	CTTCTGGTCTTTTGTATT
L158NNK-F	AAAGACCAGAAGAAGNNKTCAACCAACGGATCT
L158NNK-R	CTTCTTCTGGTCTTTTGT
T160NNK-F	CAGAAGAAGCTGTCANNKAACGGATCTTACCCA
T160NNK-R	TGACAGCTTCTTCTGGTC
N161NNK-F	AAGAAGCTGTCAACCNNKGGATCTTACCCATCA
N161NNK-R	GGTTGACAGCTTCTTCTG
G167NNK-F	GGATCTTACCCATCANNKCACACCAGTACCGGT
G167NNK-R	TGATGGGTAAGATCCGTT
R71NNK-F	ATGTACGAGAAGGGTNNKATGCTTCGCAACACA
R71NNK-R	ACCCTTCTCGTACATTGC
R74NNK-F	AAGGGTCGTATGCTTNNKAACACAGAGCGTGGA

R74NNK-R	AAGCATACGACCCTTCTC
P51NNK-F	TTAAAGTTATTGCCANNKCCGCCAGAAGTGGGG
P51NNK-R	TGGCAATAACTTTAATGA
A82NNK-F	GAGCGTGGAAAACAGNNKCAGGCTGATGCCGAT
A82NNK-R	CTGTTTTCCACGCTCTGT
D87NNK-F	GCCCAGGCTGATGCCNNKTTGGCTGCCGGTGAT
D87NNK-R	GGCATCAGCCTGGGCCTG
L88NNK-F	CAGGCTGATGCCGATNNKGCTGCCGGTGATGTC
L88NNK-R	ATCGGCATCAGCCTGGGC
A90NNK-F	GATGCCGATTTGGCTNNKGGTGATGTCGCCACT
A90NNK-R	AGCCAAATCGGCATCAGC
G162NNK-F	AAGCTGTCAACCAACNNKTCTTACCCATCAGGG
G162NNK-R	GTTGGTTGACAGCTTCTT
S163NNK-F	CTGTCAACCAACGGANNKTACCCATCAGGGCAC
S163NNK-R	TCCGTTGGTTGACAGCTT
Y164NNK-F	TCAACCAACGGATCTNNKCCATCAGGGCACACC
Y164NNK-R	AGATCCGTTGGTTGACAG
P165NNK-F	ACCAACGGATCTTACNNKTCAGGGCACACCAGT
P165NNK-R	GTAAGATCCGTTGGTTGA
S166NNK-F	AACGGATCTTACCCANNKGGGCACACCAGTACC
S166NNK-R	TGGGTAAGATCCGTTGGT
T147NNK-F	TTTGCATTCTATGGANNKGAGACTTGTAATACA
T147NNK-R	TCCATAGAATGCAAACGG
E148NNK-F	GCATTCTATGGAACCNNKACTTGTAATACAAAA
E148NNK-R	GGTTCCATAGAATGCAAA
T149NNK-F	TTCTATGGAACCGAGNNKTGTAATACAAAAGAC
T149NNK-R	CTCGGTTCCATAGAATGC
C150NNK-F	TATGGAACCGAGACTNNKAATACAAAAGACCAG
C150NNK-R	AGTCTCGGTTCCATAGAA
N151NNK-F	GGAACCGAGACTTGTNNKACAAAAGACCAGAAG
N151NNK-R	ACAAGTCTCGGTTCCATA
T152NNK-F	ACCGAGACTTGTAATNNKAAAGACCAGAAGAAG
T152NNK-R	ATTACAAGTCTCGGTTCC
K153NNK-F	GAGACTTGTAATACANNKGACCAGAAGAAGCTG
K153NNK-R	TGTATTACAAGTCTCGGT
S45NNK-F	GAACAAGCCATTGACNNKTTAAAGTTATTGCCA
S45NNK-R	GTCAATGGCTTGTTCGTT

Supplementary Table 4: Data Collection and refinement statistics

	**PhoC_1**	**PhoC_4**
Wavelength	0.976	0.976
Resolution range	50.39 - 2.24 (2.3 - 2.24)	70.51 - 1.998 (2.05 - 2.0)
Space group	P 32 2 1	P 32 2 1
Unit cell	116.38 116.38 94.54 90 90 120	117.716 117.716 97.625 90 90 120
Total reflections	970382 (42531)	1097362 (78994)
Unique reflections	49424 (3392)	53180 (3726)
Multiplicity	19.6 (12.5)	20.6 (21.2)
Completeness (%)	99.98 (100.00)	99.95 (99.73)
Mean I/sigma(I)	7.83 (0.25)	12.17 (0.37)
Wilson B-factor	48.60	46.99
R-merge	0.1681 (3.28)	0.1275 (3.874)
R-meas	0.1725 (3.418)	0.1307 (3.969)
R-pim	0.03837 (0.942)	0.02868 (0.8583)
CC1/2	0.999 (0.327)	0.999 (0.316)
CC*	1 (0.702)	1 (0.693)
Reflections used in refinement	35900 (2511)	53155 (3720)
Reflections used for R-free	1927 (130)	1936 (117)
R-work	0.2002 (0.2703)	0.1862 (0.3994)
R-free	0.2264 (0.2733)	0.2250 (0.4155)
Number of non-hydrogen atoms	5170	5462
macromolecules	5070	5277
ligands	22	51
solvent	78	134
Protein residues	663	675
RMS(bonds)	0.007	0.011
RMS(angles)	0.86	0.98
Ramachandran favored (%)	98.31	97.31
Ramachandran allowed (%)	1.54	2.10
Ramachandran outliers (%)	0.15	0.60
Rotamer outliers (%)	0.19	0.91
Clashscore	4.00	4.75
Average B-factor	63.79	58.95
macromolecules	63.93	59.29
ligands	78.14	53.67
solvent	50.50	47.93

Statistics for the highest-resolution shell are shown in parentheses.

PhoC_1 substrate scope. LC-MS chromatograms showing PhoC_1 activity towards nucleosides (**1a-32a**). Reactions were performed using nucleoside (1 mM), pyrophosphate (200 mM) and PhoC_1 (20 µM) in 20 mM sodium acetate buffer pH 4 at 30 °C.

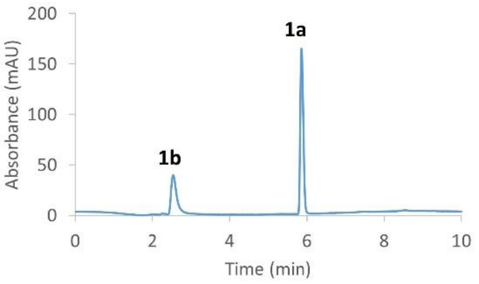

	Exp. mass (M-H$^+$)	Obs. mass
1a	324.1	324.1
1b	404.1	404.0

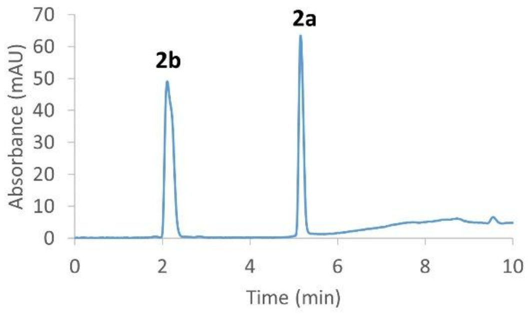

	Exp. mass (M-H$^+$)	Obs. mass
2a	266.1	266.1
2b	346.1	346.0

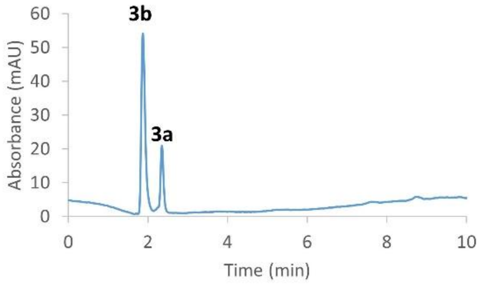

	Exp. mass (M-H$^+$)	Obs. mass
3a	282.1	282.0
3b	362.1	362.0

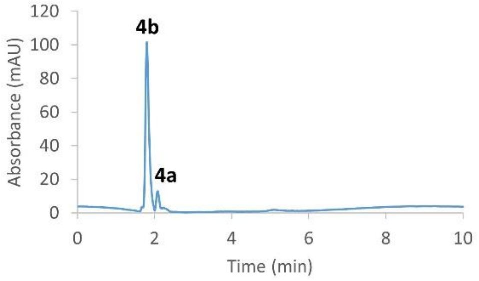

	Exp. mass (M-H$^+$)	Obs. mass
4a	242.1	242.0
4b	322.1	322.0

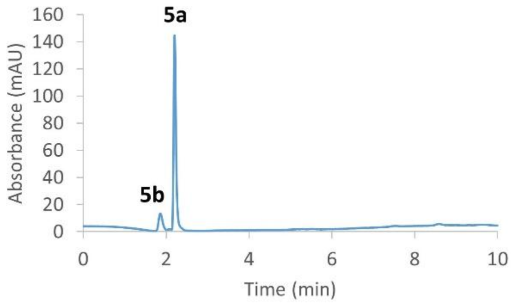

	Exp. mass (M-H$^+$)	Obs. mass
5a	243.1	243.1
5b	323.1	323.1

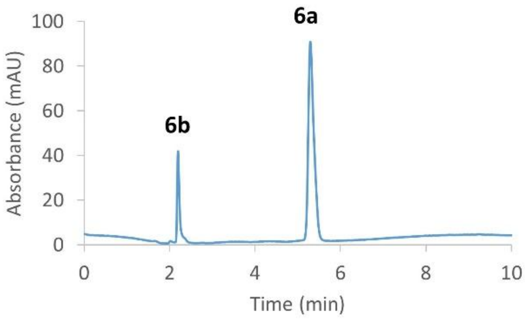

	Exp. mass (M-H$^+$)	Obs. mass
6a	250.1	250.1
6b	330.1	330.0

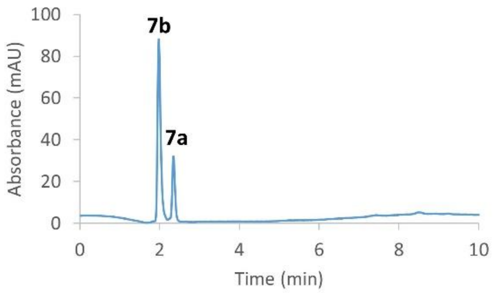

	Exp. mass (M-H⁺)	Obs. mass
7a	266.1	266.0
7b	346.1	346.0

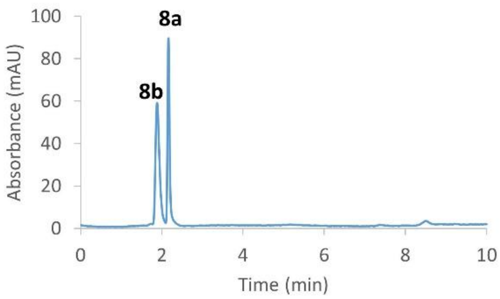

	Exp. mass (M-H⁺)	Obs. mass
8a	226.1	226.1
8b	306.1	306.1

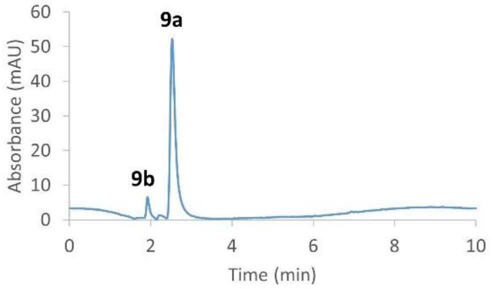

	Exp. mass (M-H⁺)	Obs. mass
9a	241.1	241.1
9b	321.1	321.1

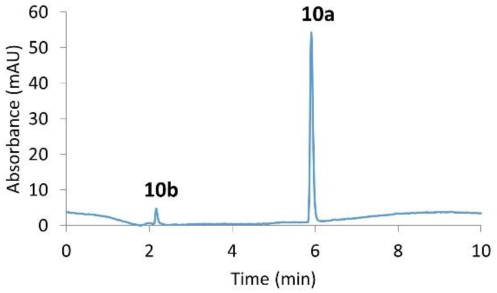

	Exp. mass (M-H⁺)	Obs. mass
10a	290.1	290.1
10b	370.1	370.1

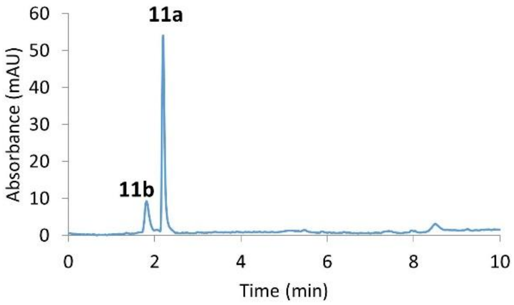

	Exp. mass (M-H⁺)	Obs. mass
11a	258.1	257.1
11b	338.1	357.1

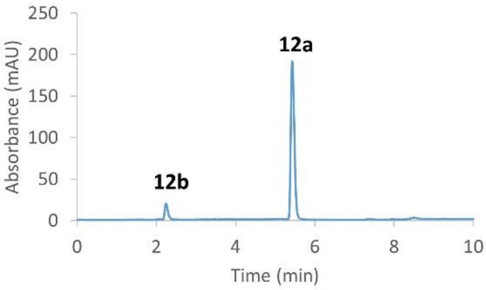

	Exp. mass (M-H⁺)	Obs. mass
12a	268.1	267.0
12b	348.1	348.1

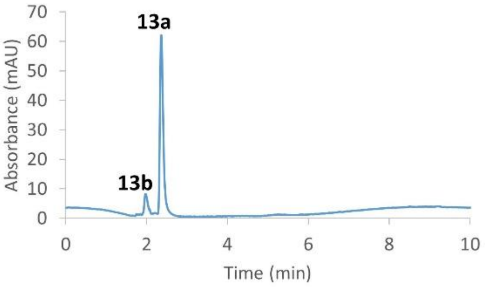

	Exp. mass (M-H⁺)	Obs. mass
13a	284.1	284.0
13b	364.1	364.0

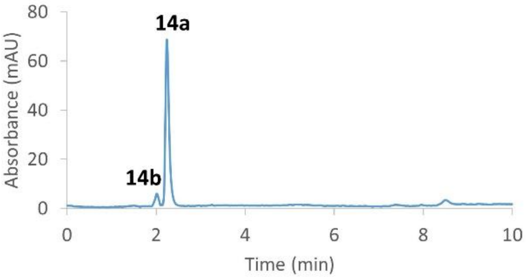

	Exp. mass (M-H⁺)	Obs. mass
14a	244.1	244.0
14b	324.1	324.0

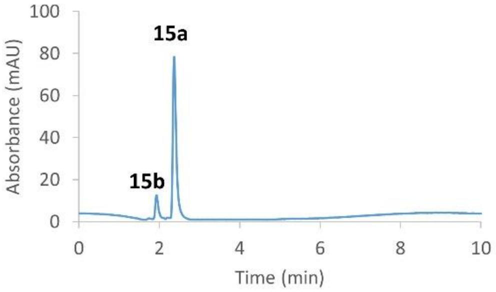

	Exp. mass (M-H⁺)	Obs. mass
15a	259.1	259.1
15b	339.1	339.1

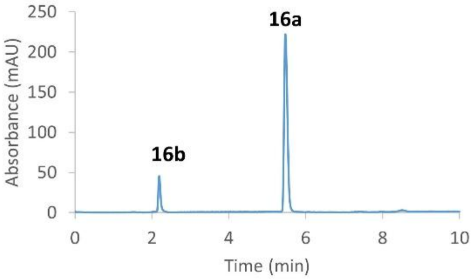

	Exp. mass (M-H⁺)	Obs. mass
16a	280.1	280.1
16b	360.1	360.1

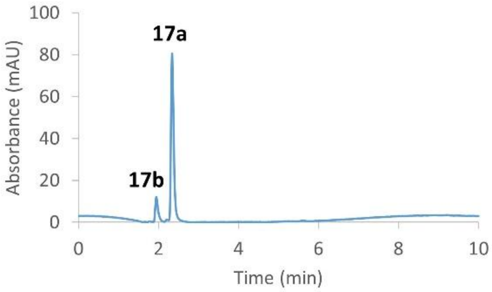

	Exp. mass (M-H⁺)	Obs. mass
17a	296.1	296.0
17b	376.1	376.0

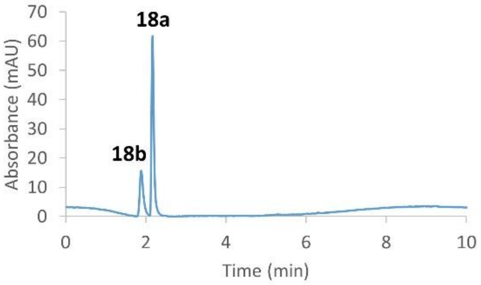

	Exp. mass (M-H⁺)	Obs. mass
18a	256.1	256.1
18b	336.1	336.1

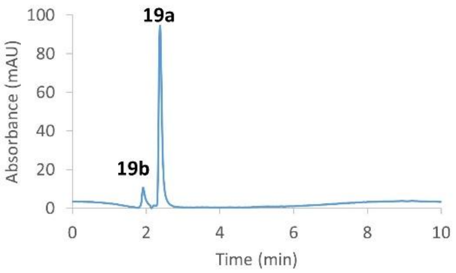

	Exp. mass (M-H+)	Obs. mass
19a	257.1	256.1
19b	337.1	336.1

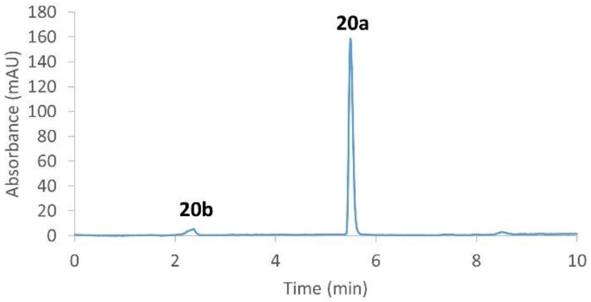

	Exp. mass (M-H+)	Obs. mass
20a	340.1	340.1
20b	420.1	420.1

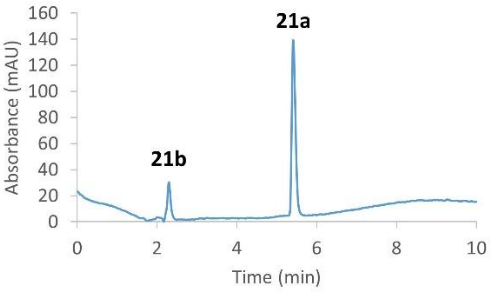

	Exp. mass (M-H+)	Obs. mass
21a	314.1	314.1
21b	394.1	394.1

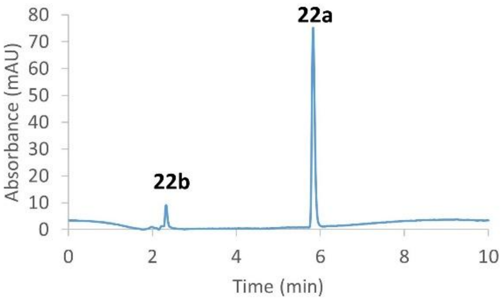

	Exp. mass (M-H+)	Obs. mass
22a	315.1	315.1
22b	395.1	395.1

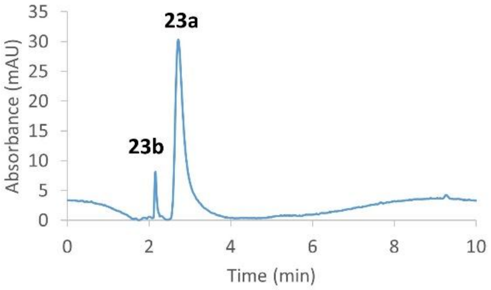

	Exp. mass (M-H+)	Obs. mass
23a	277.1	277.0
23b	357.1	356.0

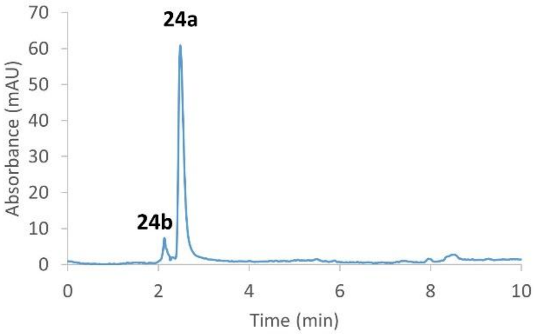

	Exp. mass (M-H+)	Obs. mass
24a	294.1	293.1
24b	374.1	373.1

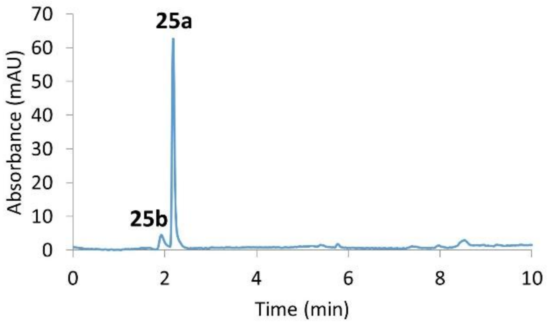

	Exp. mass (M-H+)	Obs. mass
25a	254.1	253.0
25b	334.1	332.9

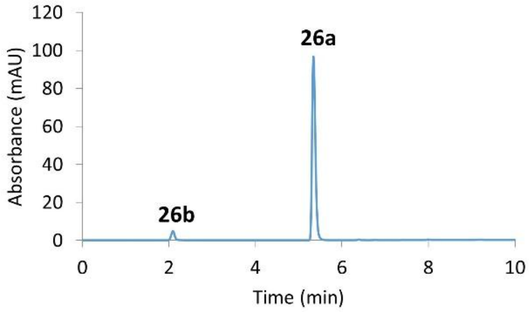

	Exp. mass (M-H+)	Obs. mass
26a	255.1	254.1
26b	335.1	334.0

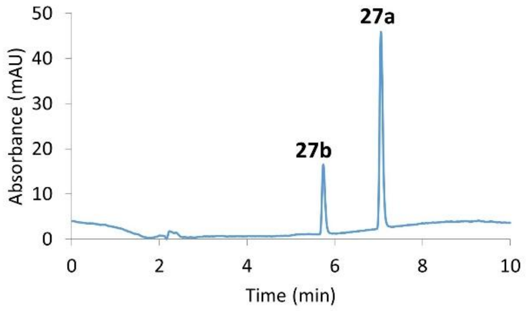

	Exp. mass (M-H+)	Obs. mass
27a	281.1	280.1
27b	361.1	360.1

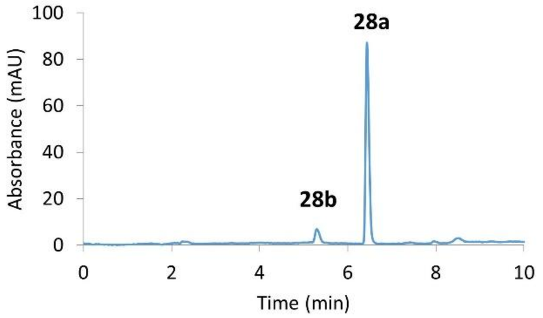

	Exp. mass (M-H+)	Obs. mass
28a	283.1	282.1
28b	363.1	362.1

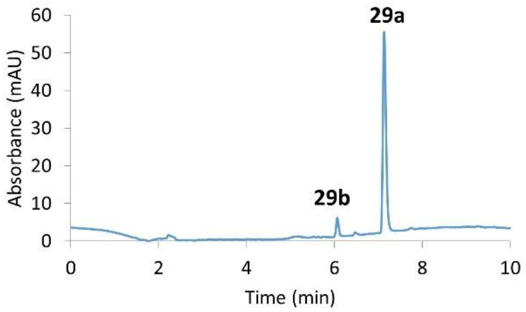

	Exp. mass (M-H+)	Obs. mass
29a	265.1	265.2
29b	345.1	345.1

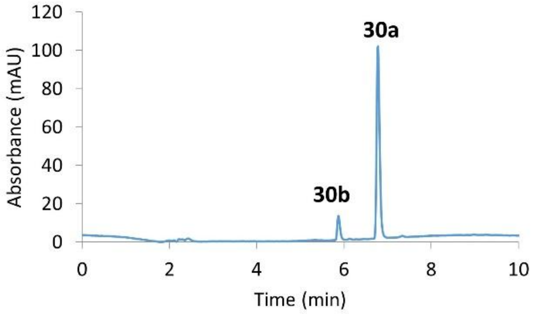

	Exp. mass (M-H+)	Obs. mass
30a	281.1	280.1
30b	361.1	360.1

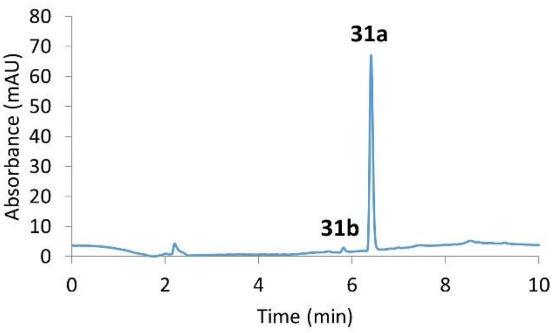

	Exp. mass (M-H+)	Obs. mass
31a	241.1	240.1
31b	321.1	320.1

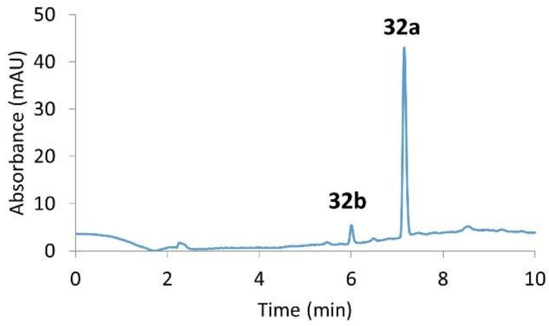

	Exp. mass (M-H+)	Obs. mass
32a	256.1	256.1
32b	336.1	336.1

PhoC_4 substrate scope. LC-MS chromatograms showing PhoC_4 activity towards nucleosides (**1a**-**32a**). Reactions were performed using nucleoside (1 mM), pyrophosphate (200 mM) and PhoC_4 (20 µM) in 20 mM sodium acetate buffer pH 4 at 30 °C

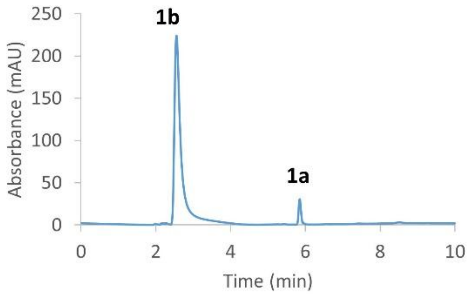

	Exp. mass (M-H+)	Obs. mass
1a	324.1	324.1
1b	404.1	404.0

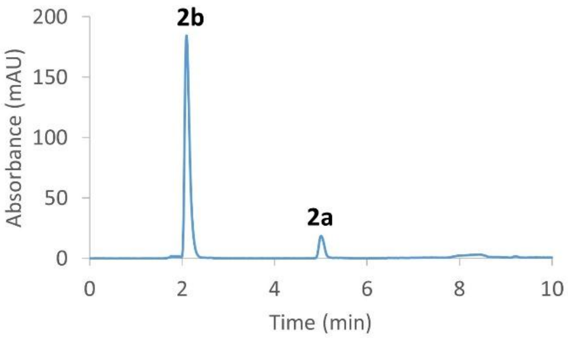

	Exp. mass (M-H+)	Obs. mass
2a	266.1	266.1
2b	346.1	346.0

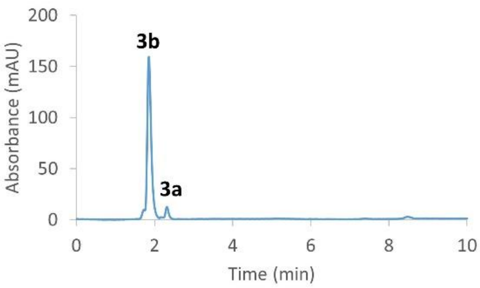

	Exp. mass (M-H+)	Obs. mass
3a	282.1	282.0
3b	362.1	362.0

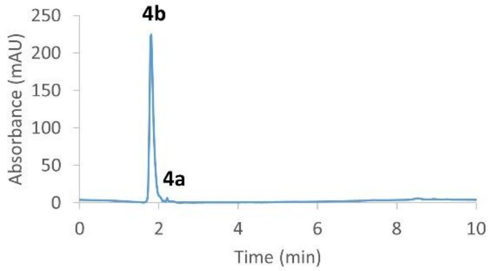

	Exp. mass (M-H+)	Obs. mass
4a	242.1	242.0
4b	322.1	322.0

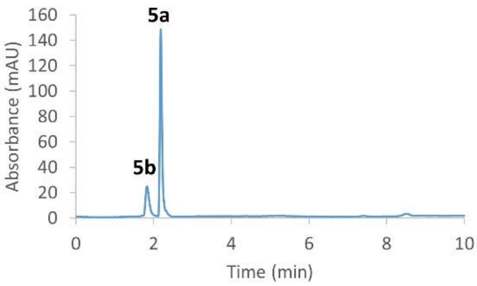

	Exp. mass (M-H$^+$)	Obs. mass
5a	243.1	243.1
5b	323.1	323.1

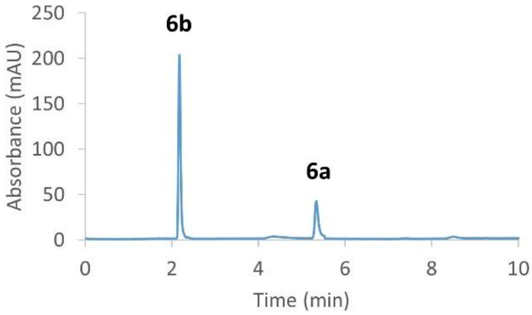

	Exp. mass (M-H$^+$)	Obs. mass
6a	250.1	250.1
6b	330.1	330.0

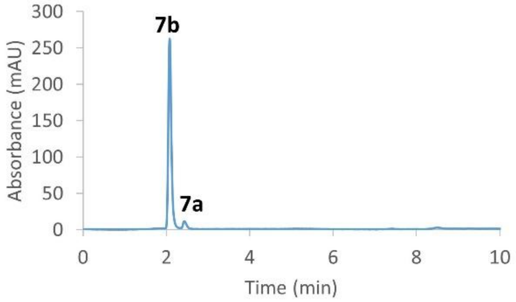

	Exp. mass (M-H$^+$)	Obs. mass
7a	266.1	266.0
7b	346.1	346.0

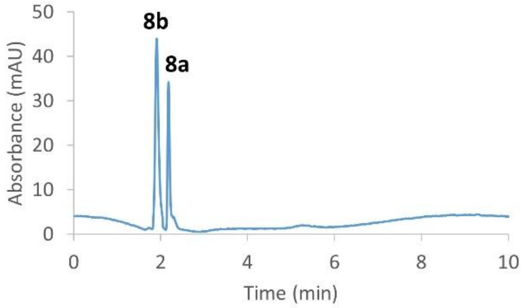

	Exp. mass (M-H$^+$)	Obs. mass
8a	226.1	226.1
8b	306.1	306.1

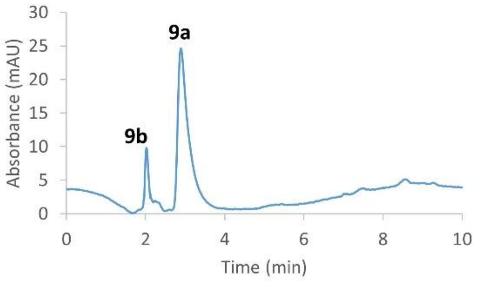

	Exp. mass (M-H$^+$)	Obs. mass
9a	241.1	241.1
9b	321.1	321.1

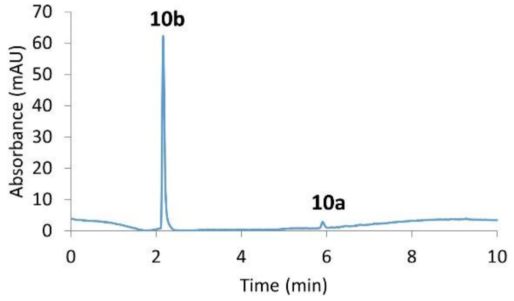

	Exp. mass (M-H$^+$)	Obs. mass
10a	290.1	290.1
10b	370.1	370.1

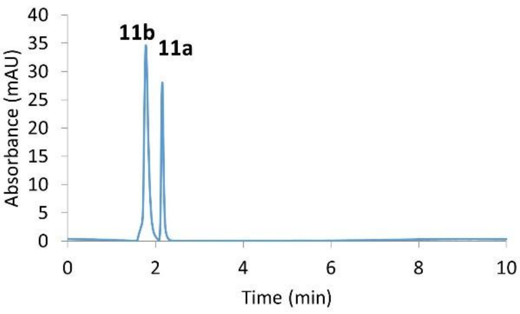

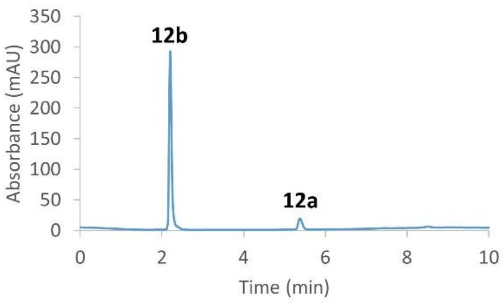

	Exp. mass (M-H$^+$)	Obs. mass
11a	258.1	257.1
11b	338.1	357.1

	Exp. mass (M-H$^+$)	Obs. mass
12a	268.1	267.0
12b	348.1	348.1

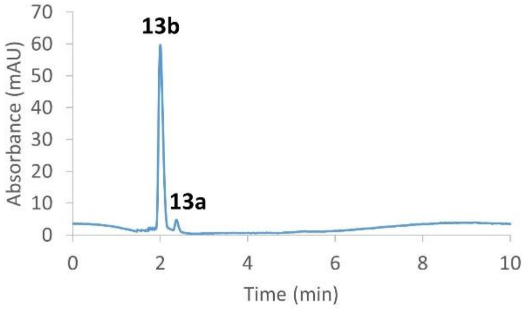

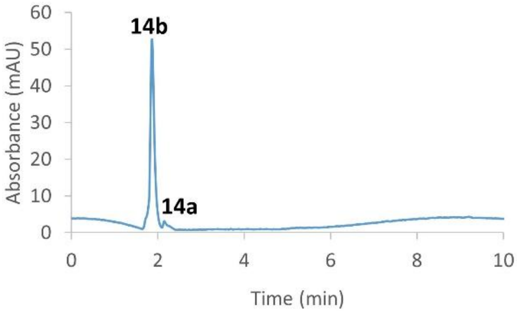

	Exp. mass (M-H$^+$)	Obs. mass
13a	284.1	284.0
13b	364.1	364.0

	Exp. mass (M-H$^+$)	Obs. mass
14a	244.1	244.0
14b	324.1	324.0

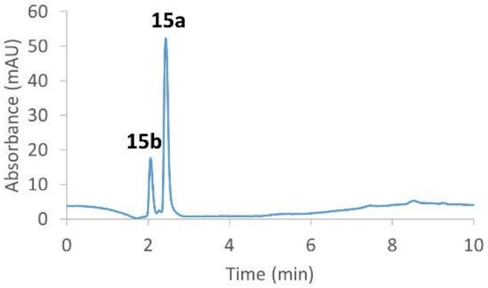

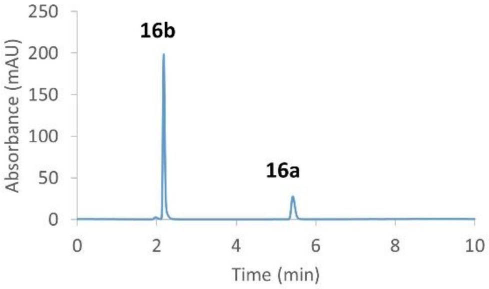

	Exp. mass (M-H$^+$)	Obs. mass
15a	259.1	259.1
15b	339.1	339.1

	Exp. mass (M-H$^+$)	Obs. mass
16a	280.1	280.1
16b	360.1	360.1

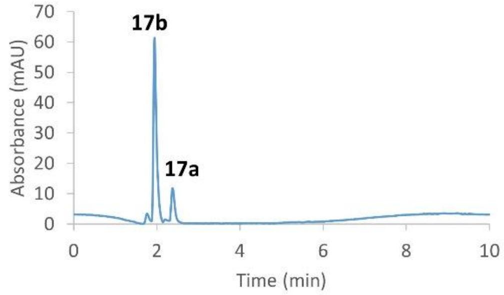

	Exp. mass (M-H$^+$)	Obs. mass
17a	296.1	296.0
17b	376.1	376.0

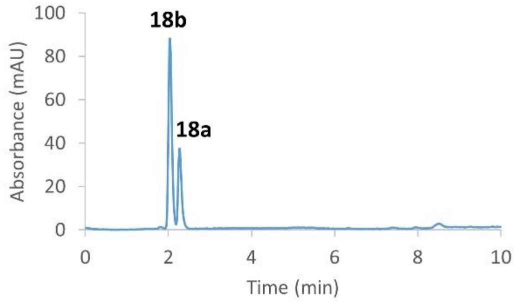

	Exp. mass (M-H$^+$)	Obs. mass
18a	256.1	256.1
18b	336.1	336.1

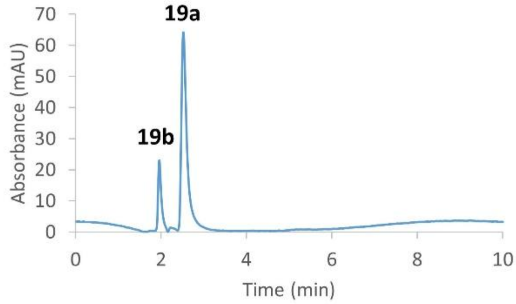

	Exp. mass (M-H$^+$)	Obs. mass
19a	257.1	256.1
19b	337.1	336.1

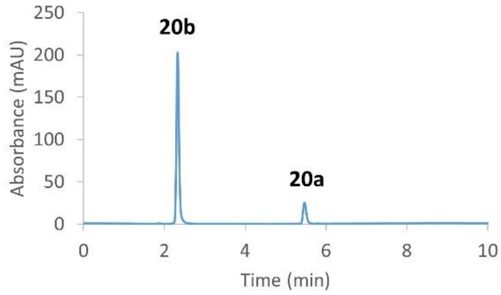

	Exp. mass (M-H$^+$)	Obs. mass
20a	340.1	340.1
20b	420.1	420.1

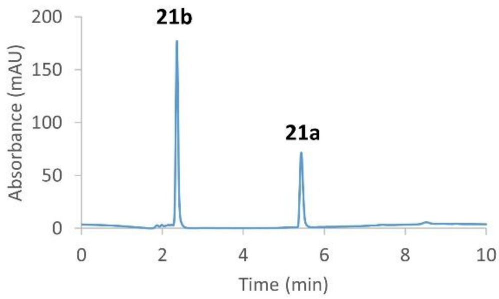

	Exp. mass (M-H$^+$)	Obs. mass
21a	314.1	314.1
21b	394.1	394.1

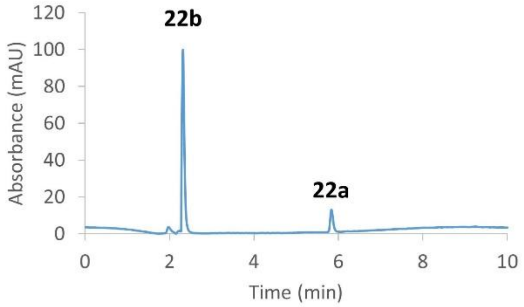

	Exp. mass (M-H$^+$)	Obs. mass
22a	315.1	315.1
22b	395.1	395.1

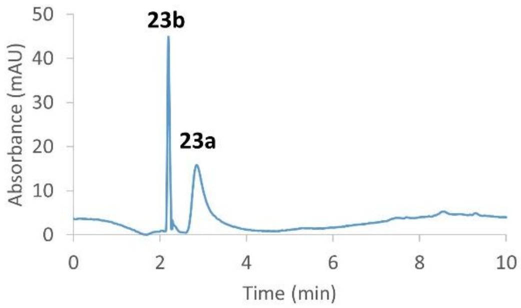

	Exp. mass ($M-H^+$)	Obs. mass
23a	277.1	277.0
23b	357.1	356.0

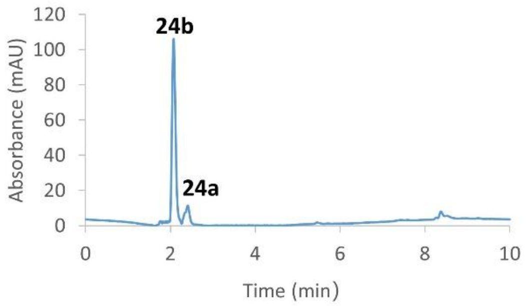

	Exp. mass ($M-H^+$)	Obs. mass
24a	294.1	293.1
24b	374.1	373.1

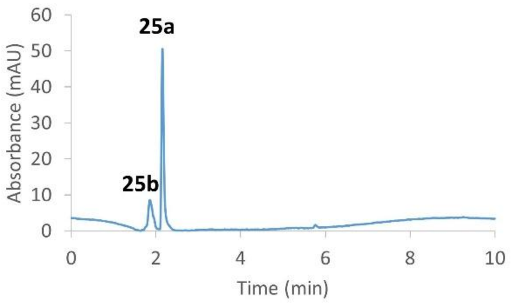

	Exp. mass ($M-H^+$)	Obs. mass
25a	254.1	253.0
25b	334.1	332.9

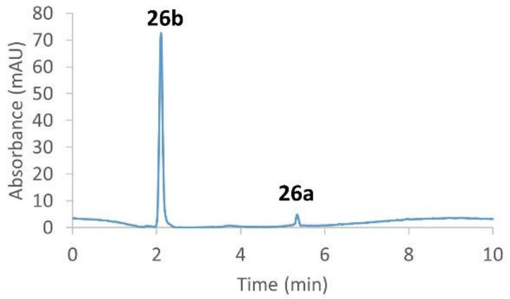

	Exp. mass ($M-H^+$)	Obs. mass
26a	255.1	254.1
26b	335.1	334.0

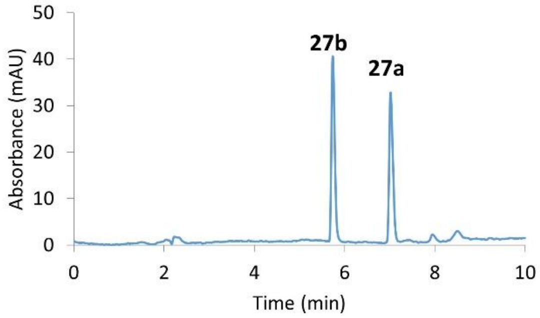

	Exp. mass ($M-H^+$)	Obs. mass
27a	281.1	280.1
27b	361.1	360.1

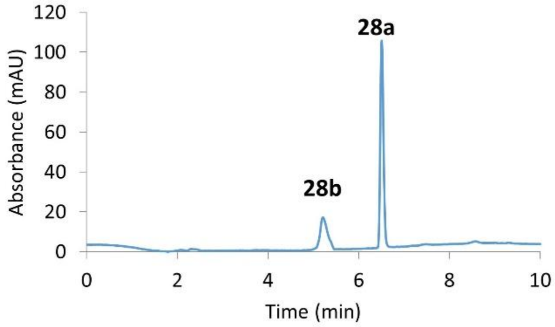

	Exp. mass ($M-H^+$)	Obs. mass
28a	283.1	282.1
28b	363.1	362.1

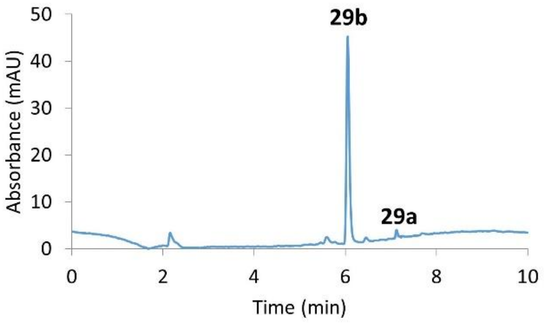

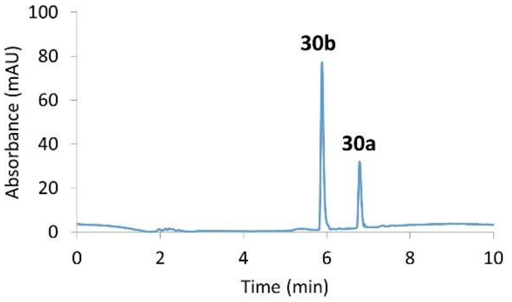

	Exp. mass (M-H$^+$)	Obs. mass
29a	265.1	265.2
29b	345.1	345.1

	Exp. mass (M-H$^+$)	Obs. mass
30a	281.1	280.1
30b	361.1	360.1

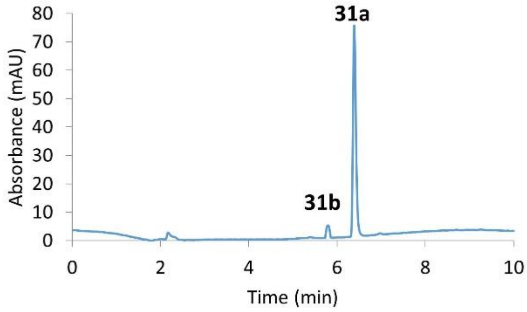

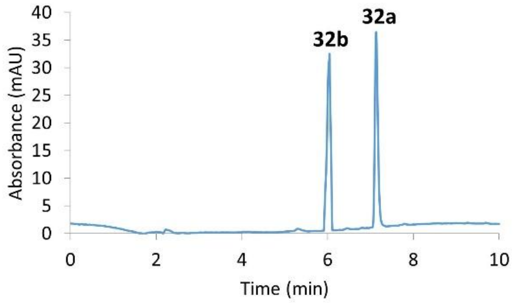

	Exp. mass (M-H$^+$)	Obs. mass
31a	241.1	240.1
31b	321.1	320.1

	Exp. mass (M-H$^+$)	Obs. mass
32a	256.1	256.1
32b	336.1	336.1

NMR spectra of chemically synthesized compounds

1b (2’-MOE-AMP)

^{1}H NMR (101 MHz, D_2O)

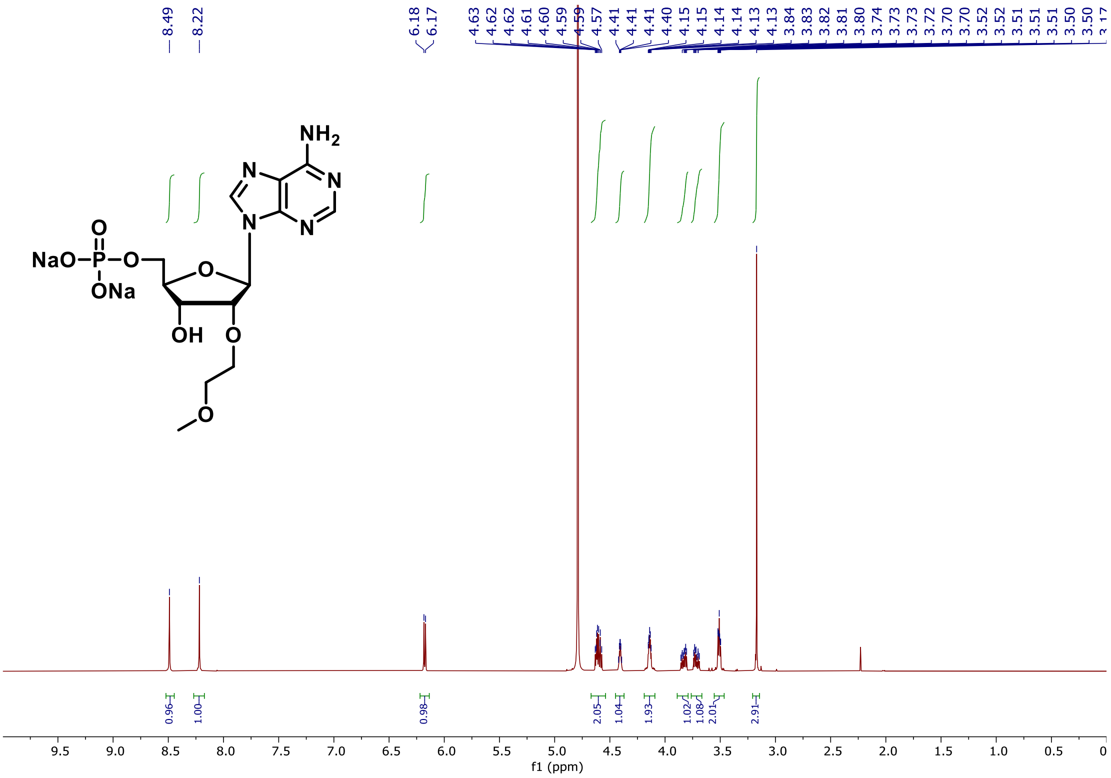

^{13}C NMR (101 MHz, D_2O)

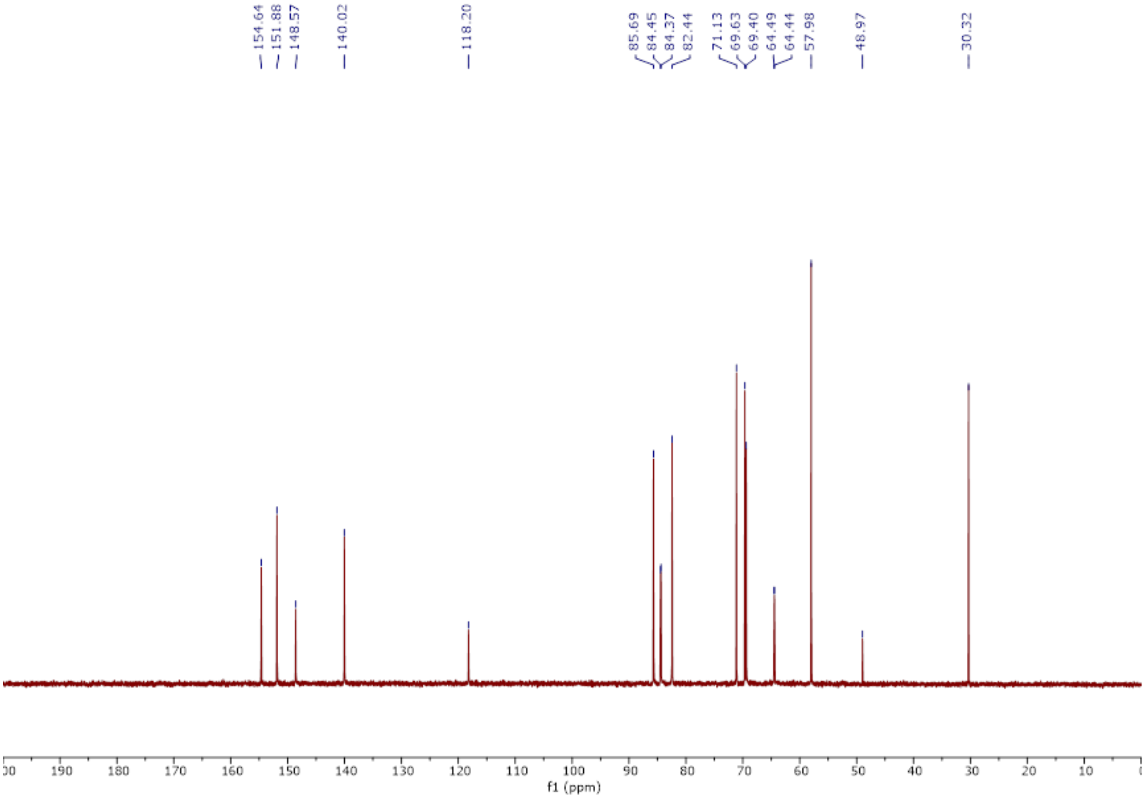

^{31}P NMR (162 MHz, D_2O)

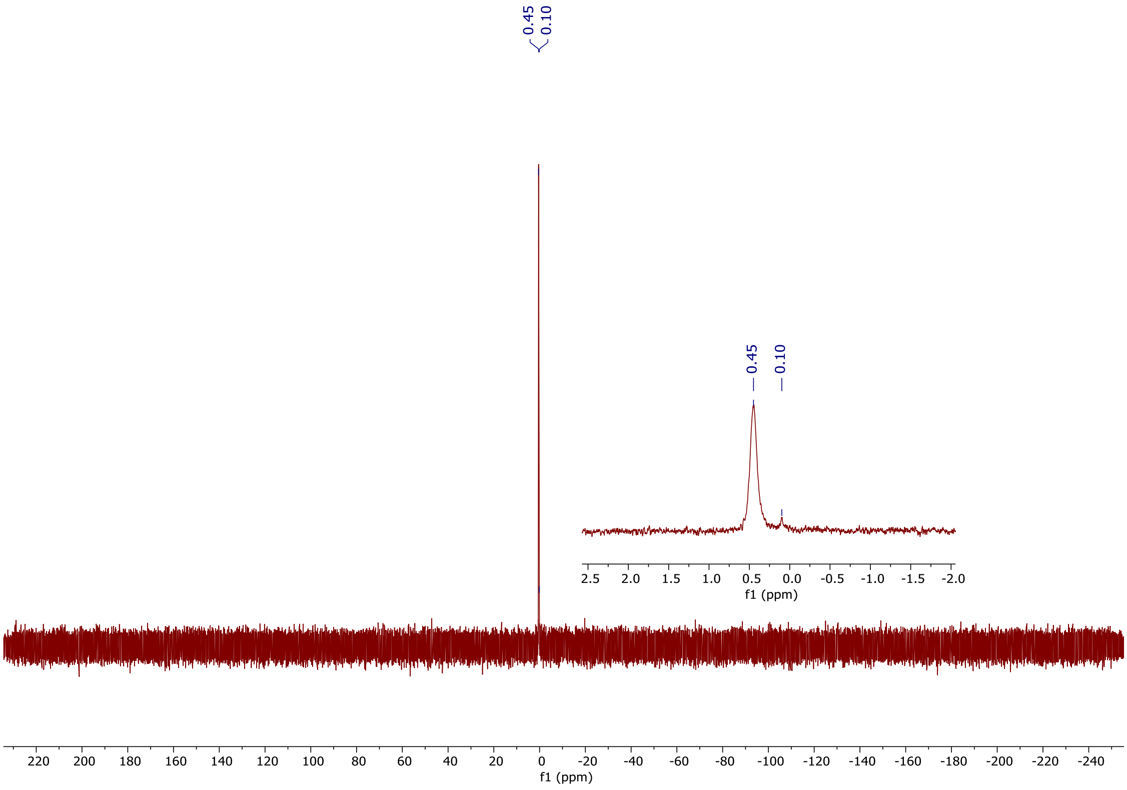

1d (2'-MOE-ATP).

^{1}H NMR (400 MHz, D_2O)

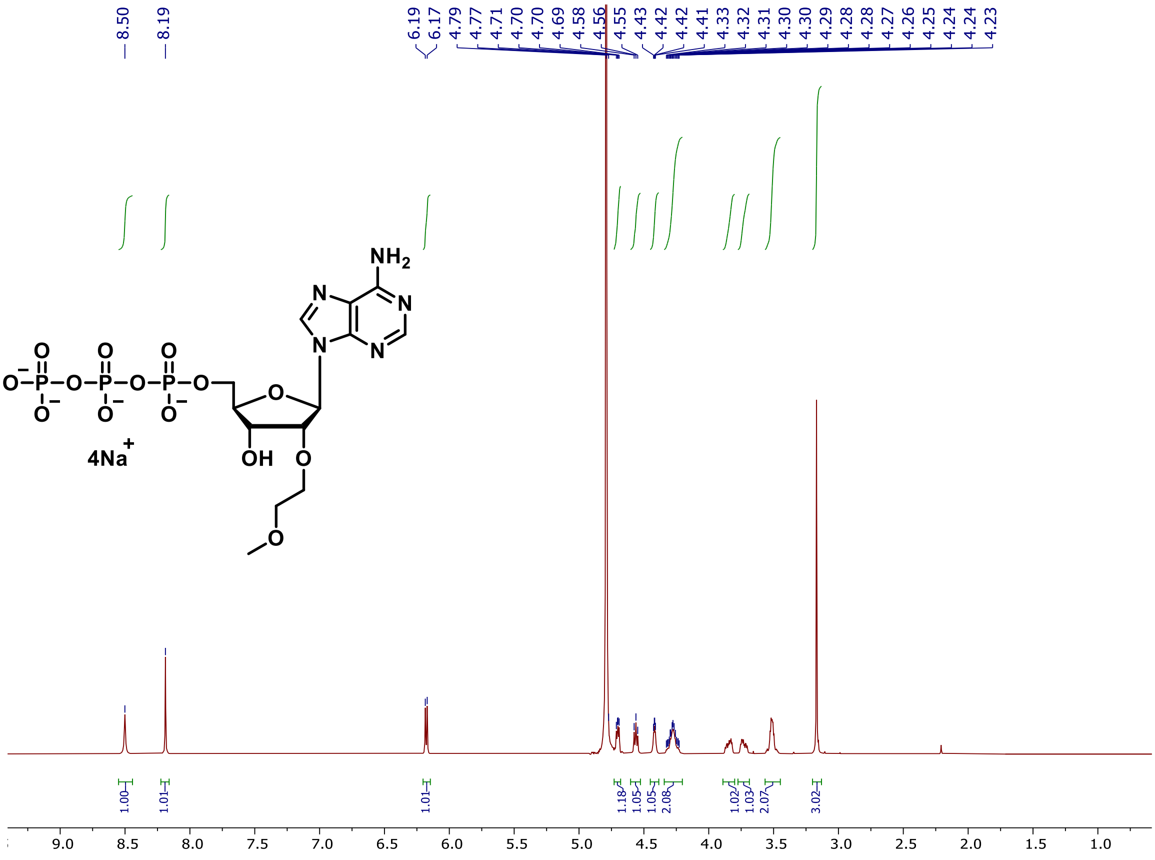

^{13}C NMR (101 MHz, D_2O)

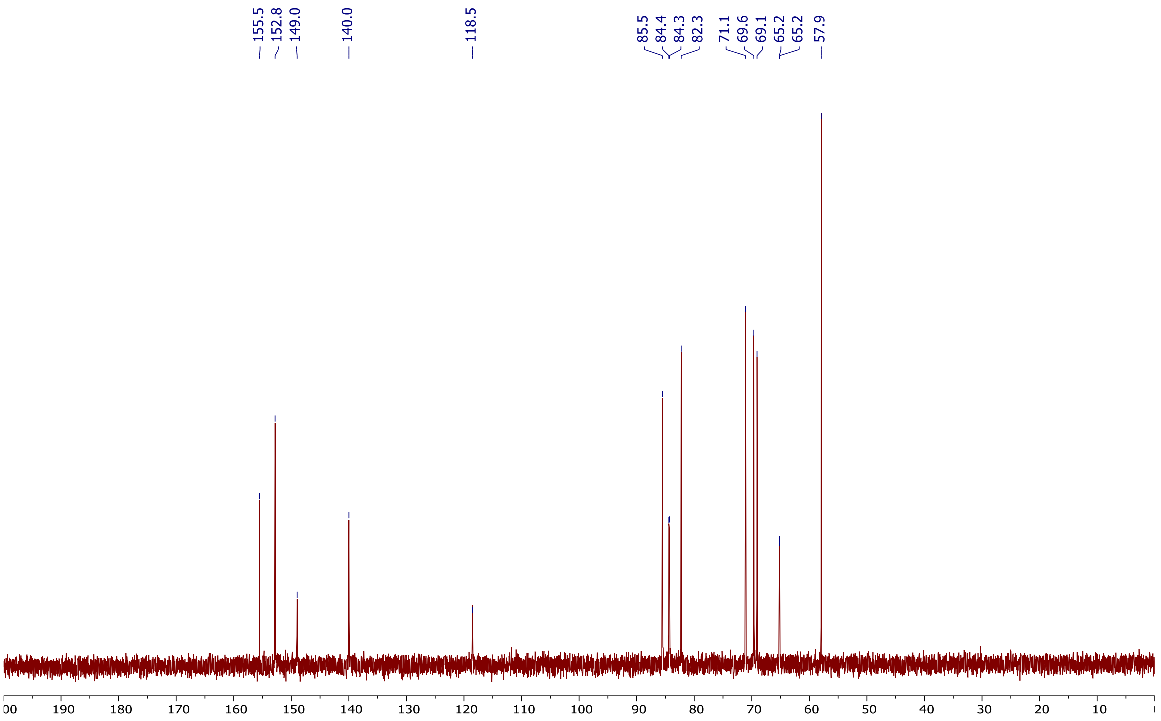

^{31}P NMR (162 MHz, D_2O)

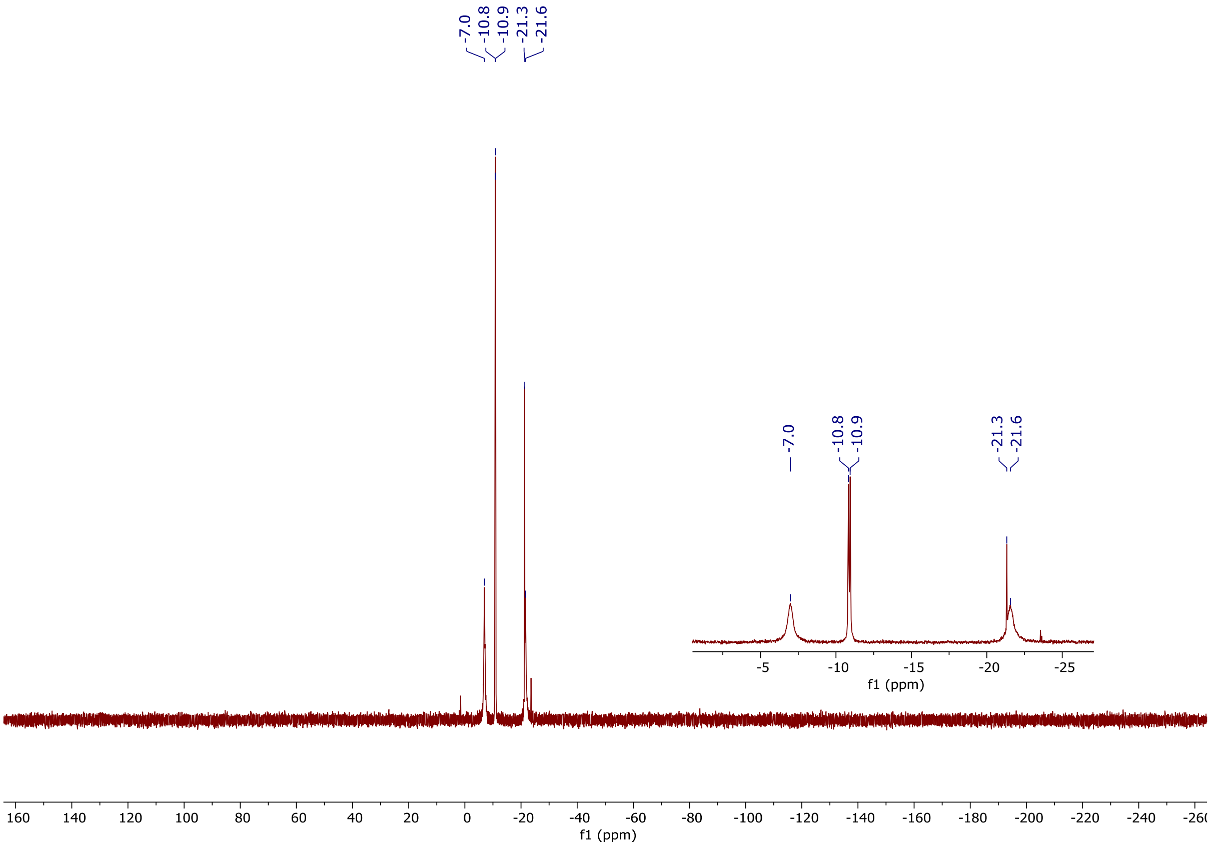

27a (3’-*O*-allyl-thymidine)

^{1}H NMR (400 MHz, $CDCl_3$)

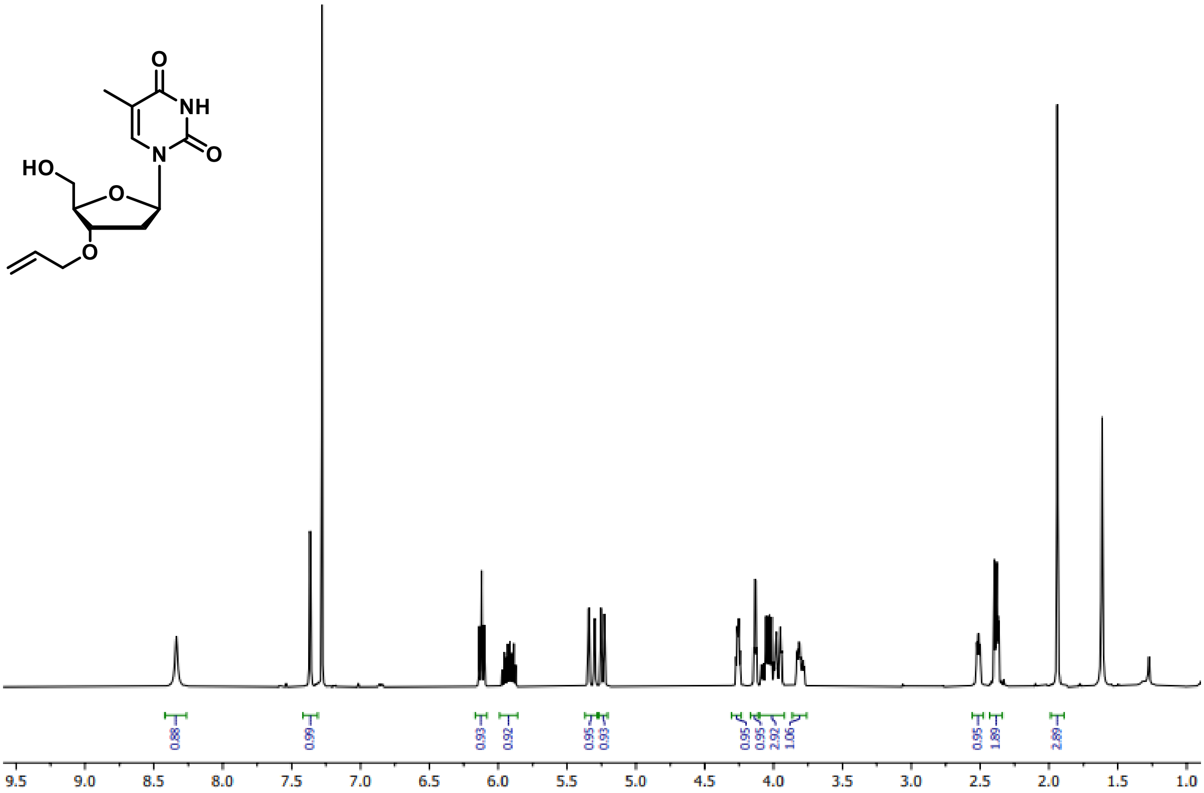

^{13}C NMR (101 MHz, $CDCl_3$)

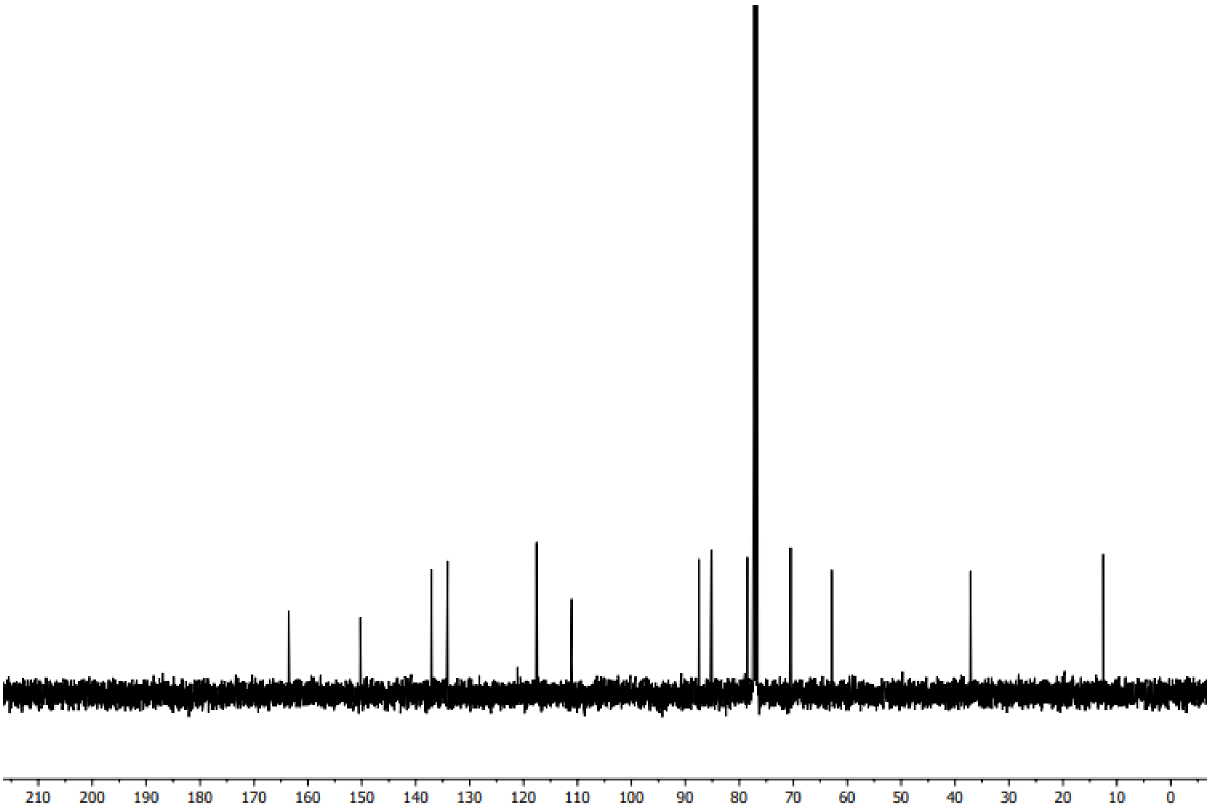

28a 3’-*O*-acetyl-thymidine

^{1}H NMR (400 MHz, $CDCl_3$)

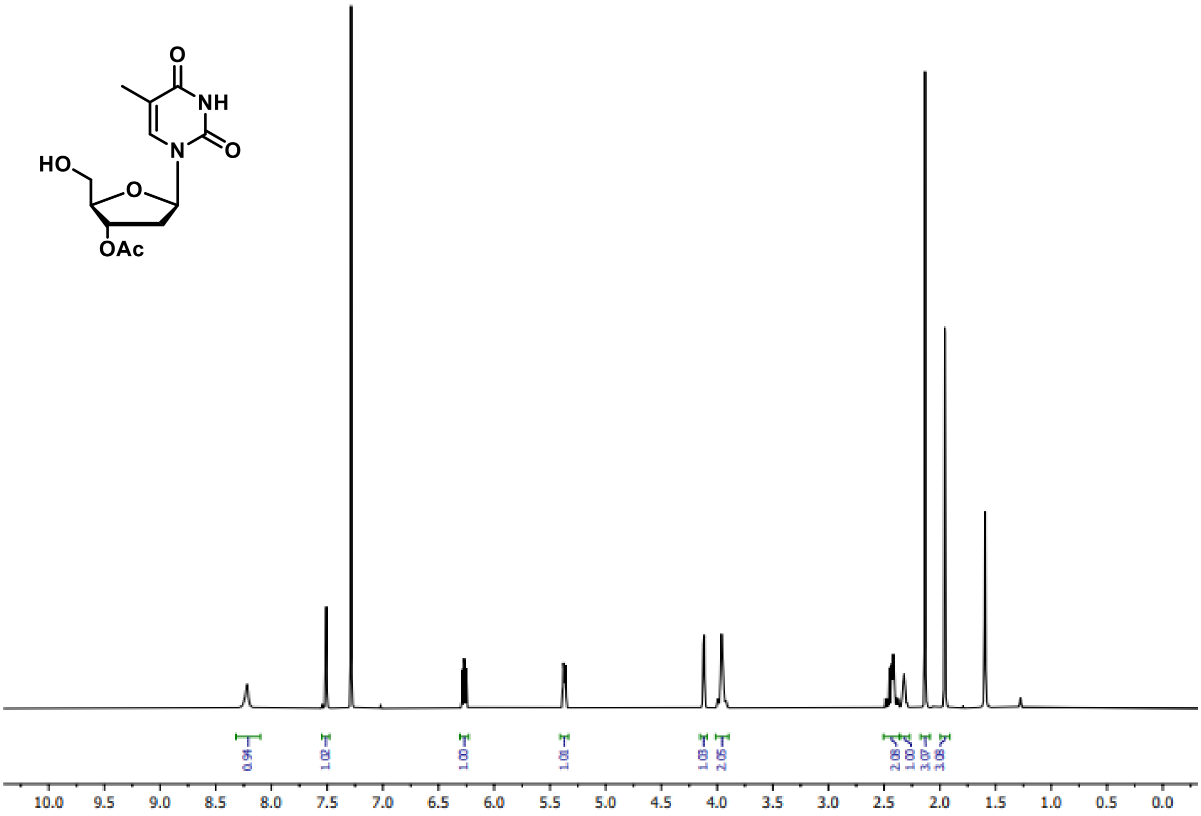

[13] C NMR (101 MHz, $CDCl_3$)

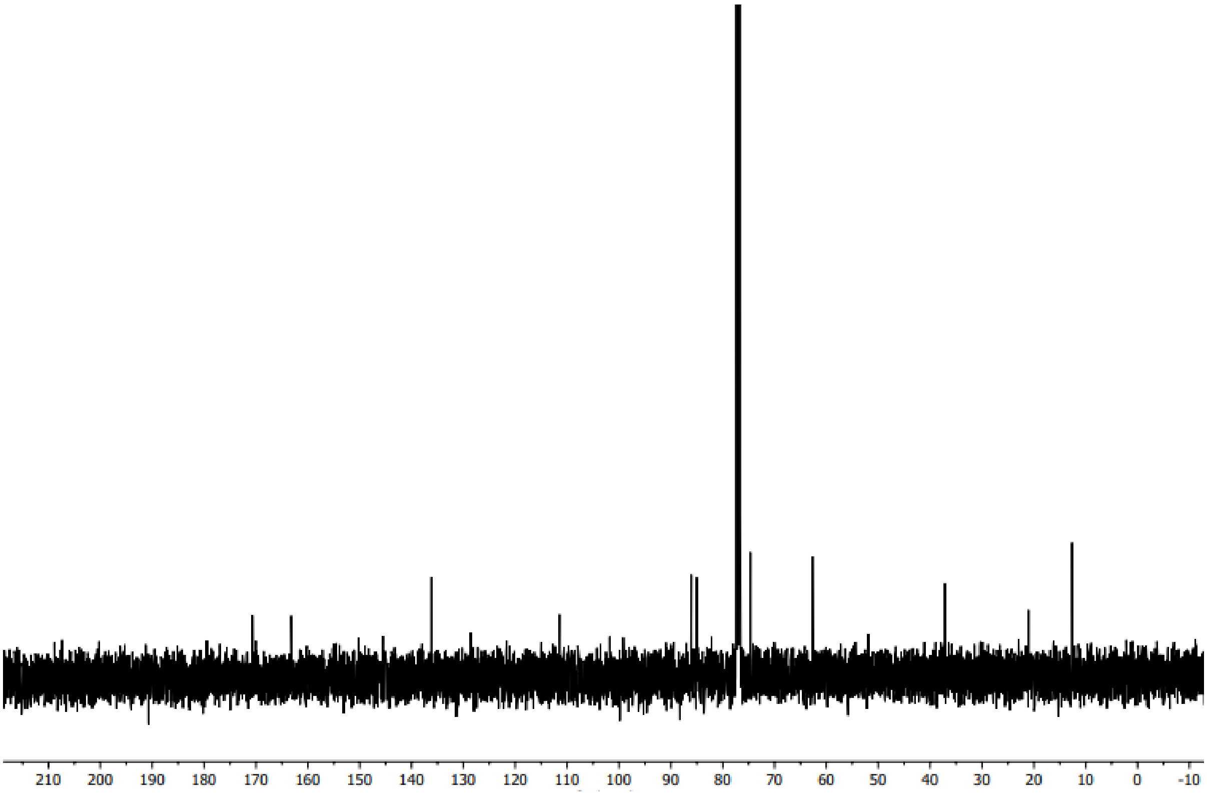

DNA and protein sequences

PhoC_4 acid phosphatase from *Morganella morganii* containing A90E, G92D, N151A, D154L and I171T mutations.

DNA sequence:

ATGAAGAAGAATATCATCGCGGGCTGCCTGTTTAGCTTATTCTCACTGAGTGCCTTAGCTGCCATTCCT
GCGGGAAACGACGCTACGACTAAGCCCGATCTTTATTATCTGAAGAACGAACAAGCCATTGACTCATT
AAAGTTATTGCCACCGCCGCCAGAAGTGGGGAGTATCCAGTTTTTAAACGACCAAGCAATGTACGAG
AAGGGTCGTATGCTTCGCAACACAGAGCGTGGAAAACAGGCCCAGGCTGATGCCGATTTGGCTGAG
GGTGATGTCGCCACTGCCTTCTCAGGCGCGTTTGGTTACCCGATCACGGAAAAAGATAGCCCCGAG
CTGTACAAGCTGTTGACAAACATGATCGAAGACGCCGGGGACCTGGCAACTCGCTCAGCAAAGGAA
CACTATATGCGCATTCGTCCGTTTGCATTCTATGGAACCGAGACTTGTGCGACAAAATTGCAGAAGAAG
CTGTCAACCAACGGATCTTACCCATCAGGGCACACCAGTACCGGTTGGGCCACAGCTCTTGTACTG
GCGGAAGTTAATCCCGCCAATCAGGACGCCATCTTAGAGCGTGGCTACCAATTAGGACAGTCCCGT
GTTATCTGCGGTTATCATTGGCAATCGGATGTCGATGCCGCACGTATCGTAGGTAGCGCTGCGGTTGC
GACCTTGCACAGTGACCCTGCATTTCAAGCGCAGCTTGCAAAGGCAAAACAAGAGTTCGCGCAAAA
GTCACAAAAA

Protein sequence:

MKKNIIAGCLFSLFSLSALAAIPAGNDATTKPDLYYLKNEQAIDSLKLLPPPPEVGSIQFLNDQAMYEKGR
MLRNTERGKQAQADADLAEGDVATAFSGAFGYPITEKDSPELYKLLTNMIEDAGDLATRSAKEHYMRIRPF
AFYGTETCATKLQKKLSTNGSYPSGHTSTGWATALVLAEVNPANQDAILERGYQLGQSRVICGYHWQSD
VDAARIVGSAAVATLHSDPAFQAQLAKAKQEFAQKSQK

PPK1 PPK from an unclassified *Erysipelotrichaceae* bacterium[1]

DNA sequence:

ATGATTAATATTTATAAAATTGATAAATTAAATAATTTTAATTTAAATAATCATAAAACTGATGATTATTCTCTTTG
TAAGGATAAAGATACAGCATTAGAATTAACACAAAAAAACATCCAAAAAATTTATGACTACCAACAAAAA
CTATATGCTGAGAAAAAAGAAGGTTTAATCATTGCTTTTCAAGCAATGGATGCTGCTGGAAAAGATGGAA
CCATTAGAGAAGTTTTAAAAGCATTAGCTCCTCAAGGCGTTCATGAAAAACCTTTTAAGTCACCAAGTTC
TACTGAATTAGCACATGATTATTTATGGAGAGTACATAATGCTGTTCCTGAAAAAGGTGAGATTACAATATT
TAATAGAAGCCATTATGAAGATGTATTAATCGGTAAAGTTAAAGAACTTTATAAATTTCAAAATAAAGCAGA
TAGAATTGATGAAAATACTGTTGTTGATAATAGATATGAAGATATTAGAAACTTTGAAAAATATCTTTATAACA
ATAGTGTAAGAATAATAAAAATATTTTTAAATGTCTCTAAAAAAGAACAAGCTGAAAGATTCCTTTCTAGAAT
AGAAGAACCAGAAAAAAATTGGAAGTTCTCTGATAGTGATTTTGAAGAAAGAGTATATTGGGATAAGTAC
CAACAAGCTTTTGAAGATGCAATAAATGCAACATCTACAAAGGATTGCCCTTGGTATGTAGTTCCTGCA
GATAGAAAATGGTATATGAGATATGTAGTATCTGAAATTGTTGTAAAAACGTTAGAAGAAATGAATCCTAAA
TATCCTACTGTTACTAAAGAAACTTTAGAAAGATTTGAAGGATATAGAACAAAACTTTTAGAAGAATACAAT
TATGATTTAGATACAATAAGACCTATAGAAAAATGA

Protein sequence:

MINIYKIDKLNNFNLNNHKTDDYSLCKDKDTALELTQKNIQKIYDYQQKLYAEKKEGLIIAFQAMDAAGKD
GTIREVLKALAPQGVHEKPFKSPSSTELAHDYLWRVHNAVPEKGEITIFNRSHYEDVLIGKVKELYKFQNK
ADRIDENTVVDNRYEDIRNFEKYLYNNSVRIIKIFLNVSKKEQAERFLSRIEEPEKNWKFSDSDFEERVYW
DKYQQAFEDAINATSTKDCPWYVVPADRKWYMRYVVSEIVVKTLEEMNPKYPTVTKETLERFEGYRTKLLE
EYNYDLDTIRPIEK

PPK2 PPK from *Cytophaga hutchinsonii*

DNA sequence:

ATGGCAACCGATTTTAGCAAACTGAGCAAATATGTTGAAACGCTGCGTGTGAAACCGAAACAGAGCAT
TGATCTGAAAAAGGATTTTGATACCGATTATGATCATAAAATGCTGACGAAAGAAGAAGGTGAAGAACTG
CTGAATCTGGGTATTAGTAAACTGAGCGAAATTCAGGAAAAACTGTATGCATCTGGCACAAAAAGCGTG
CTGATTGTTTTTCAGGCAATGGATGCAGCAGGTAAAGATGGTACCGTTAAACATATTATGACGGGTCTGA

ATCCGCAGGGTGTTAAAGTGACCAGCTTTAAAGTTCCGTCCAAAATTGAACTGAGTCATGATTATCTGTG
GCGTCATTATGTGGCACTGCCGGCAACCGGCGAAATTGGTATTTTTAACCGTAGCCATTATGAAAATGT
GCTGGTTACCCGTGTACATCCGGAATATCTGCTGAGCGAACAGACCAGCGGTGTTACCGCAATTGAA
CAGGTAAATCAGAAATTTTGGGATAAACGCTTTCAGCAGATCAATAACTTTGAACAGCATATTAGCGAAA
ACGGTACCATTGTTCTGAAATTTTTTCTGCATGTTTCCAAAAAGGAACAGAAAAAGCGTTTTATTGAACGT
ATCGAACTGGATACCAAAAATTGGAAATTTTCAACCGGTGATCTGAAAGAACGTGCCCATTGGAAAGAT
TATCGTAATGCGTATGAAGATATGCTGGCAAATACCTCTACCAAACAGGCCCCGTGGTTTGTTATTCCG
GCCGATGATAAATGGTTTACCCGTCTGCTGATTGCAGAAATTATCTGTACCGAACTGGAAAAACTGAAT
CTGACCTTTCCGACCGTGAGCCTGGAACAGAAAGCGGAACTGGAAAAAGCAAAAGCAGAACTGGTT
GCAGAAAAAATCAAGCGATCATCATCATCATCACCACTAG

Protein sequence:

MATDFSKLSKYVETLRVKPKQSIDLKKDFDTDYDHKMLTKEEGEELLNLGISKLSEIQEKLYASGTKSVLIVF
QAMDAAGKDGTVKHIMTGLNPQGVKVTSFKVPSKIELSHDYLWRHYVALPATGEIGIFNRSHYENVLVTR
VHPEYLLSEQTSGVTAIEQVNQKFWDKRFQQINNFEQHISENGTIVLKFFLHVSKKEQKKRFIERIELDTK
NWKFSTGDLKERAHWKDYRNAYEDMLANTSTKQAPWFVIPADDKWFTRLLIAEIICTELEKLNLTFPTVSL
EQKAELEKAKAELVAEKSSDHHHHH

PPK3 PPK2 variant containing A79G, S106C, I108F and L285P mutations[2]

DNA sequence

ATGGCAACCGATTTTAGCAAACTGAGCAAATATGTTGAAACGCTGCGTGTGAAACCGAAACAGAGCAT
TGATCTGAAAAAGGATTTTGATACCGATTATGATCATAAAATGCTGACGAAAGAAGAAGGTGAAGAACTG
CTGAATCTGGGTATTAGTAAACTGAGCGAAATTCAGGAAAAACTGTATGCATCTGGCACAAAAAGCGTG
CTGATTGTTTTTCAGGCAATGGATGCAGGCGGTAAAGATGGTACCGTTAAACATATTATGACGGGTCTG
AATCCGCAGGGTGTTAAAGTGACCAGCTTTAAAGTTCCGTGCAAATTTGAACTGAGTCATGATTATCTGT
GGCGTCATTATGTGGCACTGCCGGCAACCGGCGAAATTGGTATTTTTAACCGTAGCCATTATGAAAAT
GTGCTGGTTACCCGTGTACATCCGGAATATCTGCTGAGCGAACAGACCAGCGGTGTTACCGCAATTG
AACAGGTAAATCAGAAATTTTGGGATAAACGCTTTCAGCAGATCAATAACTTTGAACAGCATATTAGCGA
AAACGGTACCATTGTTCTGAAATTTTTTCTGCATGTTTCCAAAAAGGAACAGAAAAAGCGTTTTATTGAAC
GTATCGAACTGGATACCAAAAATTGGAAATTTTCAACCGGTGATCTGAAAGAACGTGCCCATTGGAAAG
ATTATCGTAATGCGTATGAAGATATGCTGGCAAATACCTCTACCAAACAGGCCCCGTGGTTTGTTATTCC
GGCCGATGATAAATGGTTTACCCGTCTGCTGATTGCAGAAATTATCTGTACCGAACTGGAAAAACTGAA
TCTGACCTTTCCGACCGTGAGCCCGGAACAGAAAGCGGAACTGGAAAAAGCAAAAGCAGAACTGGT
TGCAGAAAAAATCAAGCGATCATCATCATCATCACCACTAG

Protein sequence

MATDFSKLSKYVETLRVKPKQSIDLKKDFDTDYDHKMLTKEEGEELLNLGISKLSEIQEKLYASGTKSVLIVF
QAMDAGGKDGTVKHIMTGLNPQGVKVTSFKVPCKFELSHDYLWRHYVALPATGEIGIFNRSHYENVLVT
RVHPEYLLSEQTSGVTAIEQVNQKFWDKRFQQINNFEQHISENGTIVLKFFLHVSKKEQKKRFIERIELDTK
NWKFSTGDLKERAHWKDYRNAYEDMLANTSTKQAPWFVIPADDKWFTRLLIAEIICTELEKLNLTFPTVSP
EQKAELEKAKAELVAEKSSDHHHHH

PPK4 PPK from *Deinococcus geothermalis*

DNA sequence

ATGCAGCTCGATCGGTACCGCGTTCCCCCCGGCCAGCGGGTTCGGCTGAGCAACTGGCCCACAG
ACGACGACGGCGGCCTCAGCAAGGCCGAAGGCGAGGCGCTGCTGCCCGACCTGCAACAGCGC
CTCGCCAACCTTCAGGAGCGCCTGTACGCCGAGAGCCAGCAGGCACTCCTGATCGTATTGCAGGC
GCGTGATGCGGGCGGCAAGGACGGCACAGTCAAGCACGTGATCGGCGCCTTCAACCCGAGCGG
CGTACAGGTGAGCAATTTCAAGGTGCCGACCGAAGAGGAACGCGCCCACGACTTTCTCTGGCGCAT
CCACCGCCAGACGCCCCGCTTGGGGATGATCGGCGTCTTTAACCGCAGTCAGTATGAGGATGTCCT
GGTGACGCGTGTCCATCACCTGATCGACGACCAGACCGCCCAGCGCCGTCTGAAACACATCTGCG
CTTTCGAGTCGCTGCTCACCGACTCGGGTACACGCATTGTCAAGTTCTACCTGCACATCAGTCCTGA
GGAACAAAAGAAACGGCTCGAAGCCCGCCTGGCAGATCCCAGCAAGCACTGGAAATTCAATCCTG

GCGACCTGCAGGAACGCGCCCACTGGGACGCCTACACCGCCGTCTACGAAGACGTCTTGACGAC
CAGCACTCCCGCGGCCCCTTGGTACGTCGTTCCCGCCGACCGGAAATGGTTCCGCAACCTGCTG
GTCAGCCAGATCCTGGTCCAAACACTGGAGGAGATGAACCCACAGTTTCCGGCCCCAGCATTCAAT
GCGGCAGACCTCCGGATCGTCTAG

Protein sequence

MQLDRYRVPPGQRVRLSNWPTDDDGGLSKAEGEALLPDLQQRLANLQERLYAESQQALLIVLQARDAG
GKDGTVKHVIGAFNPSGVQVSNFKVPTEEERAHDFLWRIHRQTPRLGMIGVFNRSQYEDVLVTRVHHLI
DDQTAQRRLKHICAFESLLTDSGTRIVKFYLHISPEEQKKRLEARLADPSKHWKFNPGDLQERAHWDAYT
AVYEDVLTTSTPAAPWYVVPADRKWFRNLLVSQILVQTLEEMNPQFPAPAFNAADLRIV

PPK5 PPK from *Deinococcus radiodurans*

DNA sequence

ATGGACATTGACAACTACCGCGTCAAGCCCGGCAAACGGGTCAAACTCAGCGACTGGGCGACGAA
CGACGACGCCGGTCTGTCCAAGGAAGAAGGGCAGGCGCAGACCGCCAAACTTGCCGGCGAGCTT
GCCGAGTGGCAGGAGCGGCTCTACGCCGAGGGCAAGCAGTCGCTGCTGCTCATCTTGCAAGCGC
GCGACGCGGCGGGCAAGGACGGGGCCGTCAAAAAGGTCATCGGCGCCTTCAACCCAGCGGGC
GTGCAGATCACCAGCTTCAAGCAGCCCAGCGCCGAGGAACTCAGCCACGATTTCCTGTGGCGCAT
CCACCAGAAAGCGCCCGCCAAGGGCTATGTCGGCGTCTTCAACCGCAGCCAGTACGAGGACGTG
CTCGTCACCCGCGTCTACGACATGATCGACGACAAGACGGCCAAGCGGCGGCTGGAGCACATCC
GCCACTTCGAGGAACTGCTCACCGACAACGCCACCCGCATCGTCAAGGTCTACCTCCACATCAGC
CCGGAGGAGCAAAAGGAGCGGTTGCAGGCGCGGCTCGACAACCCCGGCAAGCACTGGAAATTCA
ACCCCGGCGACCTCAAAGACCGGAGCAACTGGGACAAATTCAACGATGTCTACGAGGACGCCCTG
ACGACCAGCACCGACGACGCGCCGTGGTACGTCGTGCCCGCCGACCGCAAGTGGTACCGCGAC
CTGGTGCTCAGCCACATTCTGCTCGGCGCGCTCAAGGACATGAATCCGCAGTTTCCGGCCATCGAC
TACGACCCGAGCAAGGTCGTCATTCACTGA

Protein sequence

MDIDNYRVKPGKRVKLSDWATNDDAGLSKEEGQAQTAKLAGELAEWQERLYAEGKQSLLLILQARDAA
GKDGAVKKVIGAFNPAGVQITSFKQPSAEELSHDFLWRIHQKAPAKGYVGVFNRSQYEDVLVTRVYDMID
DKTAKRRLEHIRHFEELLTDNATRIVKVYLHISPEEQKERLQARLDNPGKHWKFNPGDLKDRSNWDKFN
DVYEDALTTSTDDAPWYVVPADRKWYRDLVLSHILLGALKDMNPQFPAIDYDPSKVVIH

PPK6 PPK from *Treponema* sp.

DNA sequence

ATGAAAATAAAAAAATATTTTGCCGGTGATGATATTAAGTTAAAATATTTTTCGACACGGTGTGAAGAAGAT
TTGAATAAAGGTGAAGTTAAATCTATACTTATGCCTGAAAATTTAAAAAAGATGACAGACTATCAAAGCAA
ATTGTATGCGGAAGGGAAAAAAGGTTTAATTGTTGTTTTTCAGGCTATGGATACTGCCGGAAAAGACGG
CGTGATTAAACATGTTATGACTTCTTTAAATCCGCAAGGTATGTATGTTGCATCTTTTAAAGCTCCGTCCG
GTGTTGAAATGTCTCATGATTATCTTTGGCGCATTCATAAACATGCACCCTCTCGAGGCTGTGTTACGGT
TTTTAATCGCTCGCATTATGAAGATGTAATTATTGCGAGAGTACATGATTTGGTTAAAAACCAAAAACTTC
CCGATTCGATGAAGCATGACGGTATATGGAATGACCGTTACGAACAAATTCGCAATTACGAAAGTTACC
TGCATGAAAACGGTATTCATATTGTTAAATTTTTCTTGCATCTTTCAAAAGATGAGCAGAGGGAAAGGCTT
CTTTCAAGAATTGATGAGCCTGAAAAAAACTGGAAGTTTTCTAGTGCGGATATACACGAGAGAAAGTATT
GGAATGATTACCAAGATGCGTACGAAAAAGTTTTACAAAAAACTTCAACGGAAAAATCTCCATGGTATAT
AATTCCTGCTGACCAAAAATGGTTTTCACGATATTTGGTTTCAGAAATTCTTGTTGAAAAATTTAAAGACCT
TAATCCTGAGTTTCCAAAACTGCCCGAAGACGAGCTTGACAATTTGGCAAAGTGGCGTGAGGAGCTTT
T AAACGATTAG

Protein sequence

MKIKKYFAGDDIKLKYFSTRCEEDLNKGEVKSILMPENLKKMTDYQSKLYAEGKKGLIVVFQAMDTAGKDG
VIKHVMTSLNPQGMYVASFKAPSGVEMSHDYLWRIHKHAPSRGCVTVFNRSHYEDVIIARVHDLVKNQK
LPDSMKHDGIWNDRYEQIRNYESYLHENGIHIVKFFLHLSKDEQRERLLSRIDEPEKNWKFSSADIHERK

YWNDYQDAYEKVLQKTSTEKSPWYIIPADQKWFSRYLVSEILVEKFKDLNPEFPKLPEDELDNLAKWREEL
LND

PPK7 PPK from *Methylibium petroleiphilum*
DNA sequence
ATGGCCCGCTCGACCGCCTACCTGAAGACCTACCGCGTCGGCCGCAAGCTGCGGCTCAAGGACA
TCGACCCCGGCGCGCGGCCGGCCGCCAGCAGCAGCCGCGAGGCCGACGATGCGCGGCTCGC
CGAACTGGCGATCGAGATCGACCGCCTGCAGGACCTGCTCTACGCCAACGGCAGTGCCGGCCGG
CCACCCAAGCTGCTGCTGGTGCTGCAGGGCATGGACACTTCGGGCAAGGACGGCACCGCGCGCT
CTGTGTTCCGCCAGTGCAGCCCGCTCGGCGTGCGCGTGGCGGCCTTCAAGGCGCCCACCGAGG
TGGAGCGCGCGCACGACTTCCTGTGGCGCGTGCATGCGGTGGCACCGCGGGCCGGCGAGGTGG
TGGTGTTCAATCGCAGCCATTACGAAGACGTGCTGGTGCCCTTCGTCGAAGGCTGGATCGACGCCG
CCGAGCGCCAGCGTCGGCTGGCCCACATCAATGCCTTCGAGCGCCTGCTGCACGACAGCGGCA
CGACCATCGTCAAGTGCTTCCTGCACATCTCGAAGGACGAGCAGCGCGAACGGCTGCAGGCGCGT
CTGGACGACCCCGCCAAGCGCTGGAAATTCCAGGTCGGCGACCTCGAGACGCGCGAGAAGTGGA
AGGCCTACCTGGCCGCCTACGAGACCGCGCTGGCGGCCACCTCGACCGCCTGCGCGCCCTGG
CACGTGGTGCCGGCCGACAGCAAGAGCAACCGCAACCTGATGATCGCGACGCTGGTCGCGCAGG
CGCTGGCCGGCATGAAGCTCAAGCCGCCGAAGCCCGACTTCGACCCGGCGGCGGTGCGCGTGG
TCTGA
Protein sequence
MARSTAYLKTYRVGRKLRLKDIDPGARPAASSSREADDARLAELAIEIDRLQDLLYANGSAGRPPKLLLVLQ
GMDTSGKDGTARSVFRQCSPLGVRVAAFKAPTEVERAHDFLWRVHAVAPRAGEVVVFNRSHYEDVLVP
FVEGWIDAAERQRRLAHINAFERLLHDSGTTIVKCFLHISKDEQRERLQARLDDPAKRWKFQVGDLETRE
KWKAYLAAYETALAATSTACAPWHVVPADSKSNRNLMIATLVAQALAGMKLKPPKPDFDPAAVRVV

PPK8 PPK from *Meiothermus ruber*
DNA sequence
ATGAAAAAATACCGCGTTCAACCGGATGGTCGCTTTGAACTAAAGCGCTTCGATCCCGACGACACCA
GCGCCTTTGAGGGGGGCAAGCAAGCGGCCCTGGAAGCCCTGGCTGTGCTCAACAGGCGTTTGGA
GAAGCTGCAAGAGCTGCTGTATGCGGAAGGCCAGCACAAGGTACTGGTGGTGTTGCAGGCCATGGA
TGCGGGCGGCAAGGATGGCACCATCCGGGTGGTTTTCGACGGGGTAAACCCCAGCGGGGTGCGC
GTGGCCAGTTTTGGTGTGCCCACCGAGCAGGAGCTGGCCCGCGACTACCTCTGGCGGGTGCACC
AGCAGGTGCCCCGCAAGGGTGAGCTGGTGATTTTCAACCGCTCCCACTACGAGGACGTGCTGGTG
GTGCGGGTTAAAAACCTGGTGCCCCAACAGGTTTGGCAGAAGCGCTACCGCCACATCCGCGAGTT
CGAGCGCATGCTGGCCGATGAGGGAACCACCATCCTCAAATTCTTCCTGCATATCTCCAAAGACGAG
CAGCGCCAGCGGTTGCAGGAGCGCTTAGATAACCCCGAGAAGCGCTGGAAATTTCGTATGGGCGA
CCTCGAGGATCGCCGGCTTTGGGACAGGTATCAAGAGGCCTATGAAGCAGCCATCCGCGAGACCA
GCACCGAGTATGCCCCCTGGTATGTCATTCCGGCCAACAAGAACTGGTACCGCAACTGGCTGGTGA
GCCACATCCTGGTAGAAACCCTGGAGGGCTTGGCGATGCAGTACCCCCAGCCCGAAACAGCCTCG
GAGAAGATTGTGATCGAGTAG
Protein sequence
MKKYRVQPDGRFELKRFDPDDTSAFEGGKQAALEALAVLNRRLEKLQELLYAEGQHKVLVVLQAMDAG
GKDGTIRVVFDGVNPSGVRVASFGVPTEQELARDYLWRVHQQVPRKGELVIFNRSHYEDVLVVRVKNLV
PQQVWQKRYRHIREFERMLADEGTTILKFFLHISKDEQRQRLQERLDNPEKRWKFRMGDLEDRRLWDR
YQEAYEAAIRETSTEYAPWYVIPANKNWYRNWLVSHILVETLEGLAMQYPQPETASEKIVIE

PPK9 PPK from *Meiothermus silvanus*
DNA sequence
ATGGCGAAGACGATAGGAGCAACCCTAAATCTCCAGGATATCGACCCCCGCAGCACCCCCGGCTT
CAATGGGGATAAGGAAAAGGCCCTGGCCCTCCTCGAGAAGCTCACCGCCCGCCTGGACGAACTTC
AAGAGCAGCTTTACGCCGAACACCAGCACCGGGTGTTGGTGATCTTGCAGGGGATGGATACCTCTG

GCAAGGACGGCACCATCCGCCACGTGTTCAAAAACGTCGACCCGTTGGGGGTACGGGTGGTAGCG
TTCAAAGCTCCTACCCCGCCTGAGCTGGAGCGCGATTACCTGTGGCGGGTGCACCAACACGTCCC
GGCCAATGGCGAGCTGGTGATCTTCAACCGCAGCCACTACGAGGACGTGCTGGTAGCGCGGGTGC
ATAACCTGGTTCCCCCCGCAATTTGGTCGCGGCGCTACGACCACATCAATGCCTTCGAGAAGATGCT
GGTGGACGAGGGAACTACCGTGCTCAAATTTTTTTGCACATCAGCAAGGAAGAACAGAAAAAGCGC
CTCCTTGAGCGGCTCGTGGAGGCTGACAAGCACTGGAAGTTCGACCCCCAAGACCTGGTGGAGCG
GGGTTACTGGGAAGACTACATGGAAGCCTACCAGGACGTACTGGACAAGACTCACACCCAATACGC
CCCCTGGCACGTGATTCCCGCTGACCGCAAGTGGTACCGCAACCTGCAAGTCTCCCGGCTTCTGG
TCGAGGCATTAGAGGGCTTAAGGATGAAGTATCCTCGGCCCAAGCTGAATATTCCTAGGCTTAAGAGC
GAACTCGAGAAGATGTGA

Protein sequence

MAKTIGATLNLQDIDPRSTPGFNGDKEKALALLEKLTARLDELQEQLYAEHQHRVLVILQGMDTSGKDGTI
RHVFKNVDPLGVRVVAFKAPTPPELERDYLWRVHQHVPANGELVIFNRSHYEDVLVARVHNLVPPAIWS
RRYDHINAFEKMLVDEGTTVLKFFLHISKEEQKKRLLERLVEADKHWKFDPQDLVERGYWEDYMEAYQD
VLDKTHTQYAPWHVIPADRKWYRNLQVSRLLVEALEGLRMKYPRPKLNIPRLKSELEKM

Ack Acetate kinase from *Thermotoga maritima*

DNA sequence

ATGCGTGTTCTGGTTATTAATAGCGGTAGCAGCAGCATTAAATATCAGCTGATTGAAATGGAAGGTGAAA
AAGTTCTGTGTAAAGGTATTGCAGAACGTATTGGTATTGAAGGTAGCCGTCTGGTTCATCGTGTTGGTGA
TGAAAAACATGTTATTGAACGTGAACTGCCGGATCATGAAGAAGCACTGAAACTGATTCTGAATACCCT
GGTTGATGAAAAACTGGGTGTTATTAAAGATCTGAAAGAAATTGACGCAGTTGGTCATCGTGTTGTTCAT
GGTGGTGAACGTTTTAAAGAAAGCGTTCTGGTTGATGAAGAAGTTCTGAAAGCAATTGAAGAAGTTAGC
CCGCTGGCACCGCTGCATAATCCGGCAAATCTGATGGGTATTAAAGCAGCAATGAAACTGCTGCCG
GGTGTTCCGAATGTTGCAGTTTTTGATACCGCATTTCATCAGACCATTCCGCAGAAAGCATATCTGTATG
CAATTCCGTATGAATATTACGAAAAATACAAAATTCGTCGCTACGGTTTTCATGGTACCAGCCATCGTTAT
GTTAGCAAACGTGCAGCAGAAATTCTGGGTAAAAAACTGGAAGAACTGAAAATTATCACCTGTCATATT
GGTAATGGTGCAAGCGTTGCAGCAGTTAAATATGGTAAATGTGTTGATACCAGCATGGGTTTTACCCCG
CTGGAAGGTCTGGTTATGGGTACCCGTAGCGGTGATCTGGATCCGGCAATTCCGTTTTTTATTATGGAA
AAAGAGGGTATTAGCCCGCAGGAAATGTATGATATTCTGAATAAAAAAAGCGGCGTTTATGGTCTGAGC
AAAGGTTTTAGCAGCGATATGCGTGATATTGAAGAAGCAGCACTGAAAGGTGATGAATGGTGTAAACTG
GTTCTGGAAATTTATGATTACCGTATTGCAAAATACATCGGTGCATACGCTGCAGCAATGAATGGTGTTG
ATGCAATTGTTTTTACCGCAGGTGTTGGTGAAAATAGCCCGATTACCCGTGAAGATGTTTGTAGCTATCT
GGAATTTCTGGGTGTTAAACTGGATAAACAGAAAAATGAAGAcACCATTCGTGGTAAAGAAGGTATTATT
AGCACCCCGGATAGCCGTGTTAAAGTTCTGGTTGTTCCGACCAATGAAGAACTGATGATTGCACGTGA
TACCAAAGAAATTGTTGAAAAAATCGGTCGTTAA

Protein sequence

MRVLVINSGSSSIKYQLIEMEGEKVLCKGIAERIGIEGSRLVHRVGDEKHVIERELPDHEEALKLILNTLVDE
KLGVIKDLKEIDAVGHRVVHGGERFKESVLVDEEVLKAIEEVSPLAPLHNPANLMGIKAAMKLLPGVPNVA
VFDTAFHQTIPQKAYLYAIPYEYYEKYKIRRYGFHGTSHRYVSKRAAEILGKKLEELKIITCHIGNGASVAAVK
YGKCVDTSMGFTPLEGLVMGTRSGDLDPAIPFFIMEKEGISPQEMYDILNKKSGVYGLSKGFSSDMRDIE
EAALKGDEWCKLVLEIYDYRIAKYIGAYAAAMNGVDAIVFTAGVGENSPITREDVCSYLEFLGVKLDKQKN
EETIRGKEGIISTPDSRVKVLVVPTNEELMIARDTKEIVEKIGR

Supplementary References:

1. Tavanti, M., Hosford, J., Lloyd, R. C. & Brown, M. J. B. ATP regeneration by a single polyphosphate kinase powers multigram-scale aldehyde synthesisin vitro. *Green Chem.* **23**, 828–837 (2021).
2. Shen, Q. et al. Semirational engineering of *Cytophaga hutchinsonii* polyphosphate kinase

for developing a cost-effective, robust, and efficient adenosine 5'-triphosphate regeneration system. *Appl. Environ. Microbiol.* **89**, 1–20 (2023).
